# Time course transcriptomic profiling suggests Crp/Fnr transcriptional regulation of *nosZ* gene in a N_2_O-reducing thermophile

**DOI:** 10.1016/j.isci.2024.111074

**Published:** 2024-09-30

**Authors:** Jiro Tsuchiya, Sayaka Mino, Fuki Fujiwara, Nao Okuma, Yasunori Ichihashi, Robert M. Morris, Brook L. Nunn, Emma Timmins-Schiffman, Tomoo Sawabe

**Affiliations:** 1 Faculty of Fisheries Sciences, Hokkaido University, Hakodate, Hokkaido, Japan; 2 RIKEN BioResource Research Center, Tsukuba, Ibaraki, Japan; 3 Graduate School of Agricultural and Life Sciences, The University of Tokyo, Bunkyo-ku, Tokyo, Japan; 4 School of Oceanography, University of Washington, Seattle, WA, USA; 5 Department of Genome Sciences, University of Washington, Seattle, WA, USA

**Keywords:** Aquatic biology, Microbiology, Molecular microbiology, Omics, Transcriptomics

## Abstract

*Nitrosophilus labii* HRV44^T^ is a thermophilic chemolithoautotroph possessing clade II type nitrous oxide (N_2_O) reductase (NosZ) that has an outstanding activity in reducing N_2_O to dinitrogen gas. Here, we attempt to understand molecular responses of HRV44^T^ to N_2_O. Time course transcriptome and proteomic mass spectrometry analyses under anaerobic conditions revealed that most of transcripts and peptides related to denitrification were constitutively detected, even in the absence of any nitrogen oxides as electron acceptors. Gene expressions involved in electron transport to NosZ were upregulated within 3 h in response to N_2_O, rather than upregulation of *nos* genes. Two genes encoding Crp/Fnr transcriptional regulators observed upstream of *nap* and *nor* gene clusters had significant negative correlations with *nosZ* expression. Statistical path analysis further inferred a significant causal relationship between the gene expression of *nosZ* and that of one Crp/Fnr regulators. Our findings contribute to understanding the transcriptional regulation in clade II type N_2_O-reducers.

## Highlights

• Denitrification genes are constitutively expressed under anaerobic conditions
• Negative regulation of transcriptional regulator on expression of *nosZ* was inferred
• Electron-transport activity to NosZ may be increased in response to N_2_O

## Graphical abstract

**Figure:**
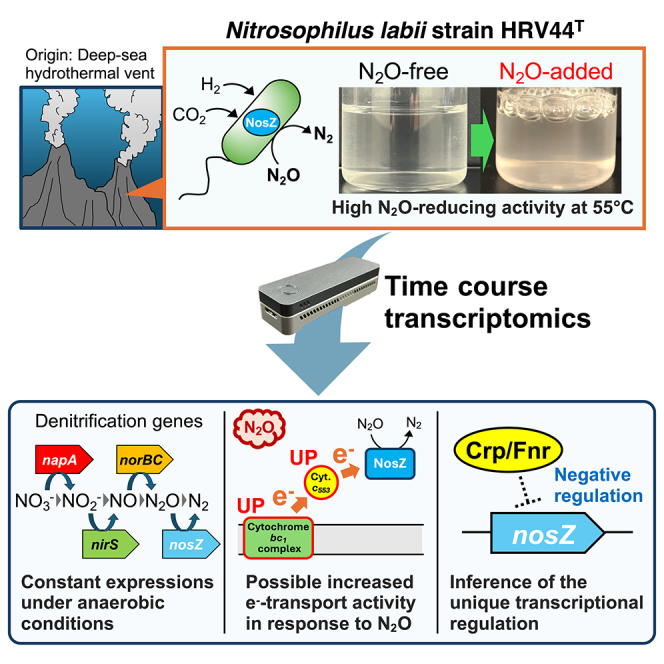

## Introduction

Nitrous oxide (N_2_O) is a colorless, odorless, and inert gas with an atmospheric lifetime of 109 years. It has recently gained attention as a cause of serious ozone depletion and a potent greenhouse gas that has a global warming potential (GWP) 273 times higher than CO_2_.^1^^,^^2^ The concentration of atmospheric N_2_O has increased by more than 20% since the 18^th^ century,^3^ with large N_2_O emissions coming from agricultural lands, natural soils, and oceans. In both terrestrial and aquatic ecosystems, N_2_O production is mainly triggered by microbial nitrogen metabolisms, particularly nitrification and denitrification. While denitrification can lead to the emission of N_2_O, the final step of denitrification (N_2_O + 2H^+^ + 2e^−^ → N_2_ + H_2_O) is the pathway reducing N_2_O to harmless dinitrogen gas (N_2_). This reaction is the only known process for the biological removal of N_2_O in natural environments^4^ and has been thoroughly investigated due to its ecological significance and potential application for bioremediation.

Microbial N_2_O reduction is primarily mediated by N_2_O reductase (NosZ) and by accessory Nos proteins encoded by *nos* genes. The key enzyme gene, *nosZ*, is phylogenetically divided into two clades that differ in the apo-NosZ translocation system across the cell membrane and partially in the structure of the accessory *nos* genes.^4^^,^^5^ Clade Ⅰ NosZ uses the Tat system and clade Ⅱ NosZ uses the Sec system, with several exceptions. In addition to variations in the *nos* gene cluster, clade Ⅱ type N_2_O-reducing microorganisms, which possess a higher affinity for N_2_O than clade I type N_2_O-reducers,^6^ have been found in diverse microbial taxa and are abundant in both terrestrial and marine environments.^7^ These reports suggest that clade Ⅱ type N_2_O-reducers are particularly important in curbing N_2_O emission in nature. However, there are relatively few detailed studies of clade II type *nosZ* genes, and the remarkable taxonomical diversity in clade Ⅱ type N_2_O-reducers highlights the need to enhance understanding of their functions and roles in environmental N_2_O reduction.^8^

Chemolithoautotrophs are the primary producers in deep-sea hydrothermal systems (e.g., Jørgensen and Boetius^9^). Members of the class “*Campylobacteria*” (homotypic synonym of *Epsilonproteobacteria*) are often the predominant bacteria in these systems^10^^,^^11^^,^^12^ and have the ability to utilize a broad range of inorganic compounds for energy. Some species of “*Campylobacteria*” isolated from deep-sea hydrothermal systems possess the periplasmic nitrate reductase gene (*nap*) and *nos* gene clusters, indicating the genetic potential for denitrification.^13^^,^^14^ Since the development of a cultivation method for N_2_O-reducing chemolithoautotrophs,^13^ thermophilic “*Campylobacteria*” belonging to genera *Nitrosophilus* and *Nitratiruptor* have been found to grow by utilizing exogenous N_2_O as the sole electron acceptor.^14^^,^^15^ It also has been shown that deep-sea hydrothermal vent “*Campylobacteria*” species have clade Ⅱ type *nosZ* genes.^14^ *Nitrosophilus labii* HRV44^T^ is a thermophilic “*Campylobacteria*” isolated from a deep-sea hydrothermal vent in the Mid-Okinawa Trough, Japan, with the highest N_2_O-reducing activity observed among its related species.^14^ However, the molecular mechanisms regulating the N_2_O-reduction in deep-sea hydrothermal vent “*Campylobacteria*” have not been investigated.

Physiological responses to nitrogen oxides and patterns of transcriptional regulation for denitrification genes have been studied in several N_2_O-reducing bacteria by transcriptome and protein analyses. For example, previous study in *Pseudomonas stutzeri*, which has a clade Ⅰ *nosZ*, found that N_2_O is a weak inducer of *nosZ* expression but that nitrogen oxide (NO) induces the transcriptional regulator, dissimilative nitrate respiration regulator D (DnrD), which leads to higher levels of *nosZ* transcription.^16^ A study of *Paracoccus denitrificans*, which also has a clade Ⅰ *nosZ*, indicated that its *nosZ* transcription is regulated by oxygen depletion or NO via fumarate and nitrate reduction (Fnr) transcriptional regulator or nitric oxide reductase regulator (NNR), respectively.^17^ *Wolinella succinogenes* is a model “*Campylobacteria*” with a clade Ⅱ type *nosZ*. NapA and NosZ proteins in *W*. *succinogenes* were upregulated in wild-type cells when nitrate and N_2_O were supplied as terminal electron acceptors, respectively, possibly due to the regulation by transcriptional regulators of neurotransmitter:sodium symporter (NSS) family NssA, NssB, and NssC, which belong to the Crp/Fnr superfamily protein.^18^

RNA sequencing (RNA-seq) enables genome-wide gene expression analysis in model organisms. Nanopore-based RNA-seq has been increasingly used in eukaryotes, including mammalians, plants, insects, and crustaceans (e.g., Wang et al.,^19^ Bayega et al,^20^ and Wang et al.^21^). However, its application in prokaryotes has yet to be fully evaluated, and this technology exhibits a relatively high error rate on raw sequences compared to standard next-generation sequencing (NGS) devices such as Illumina. Still, Nanopore-based RNA-seq with amplification of cDNA has an advantage in enabling library preparation from small amounts of RNA extracted from low-growth microorganisms (e.g., cells that are hard to culture or grown under non-optimal growth conditions). Thus, this platform can be ideal for understanding transcriptomic traits of microorganisms that are difficult to recover sufficient amounts of RNA for conventional Illumina RNA-seq, such as chemolithoautotrophs in deep-sea hydrothermal environments, where their cell densities are often low even under optimal growth conditions.

Here, we conduct RNA-seq with a single-molecule long-read sequencing platform and proteome analysis to investigate gene and protein expression in response to N_2_O in *Nitrosophilus labii* HRV44^T^, a model organism of N_2_O-reducing bacteria isolated from a deep-sea hydrothermal vent.

## Results

### Cell growth and N_2_O consumption dynamics

**Figure 1:**
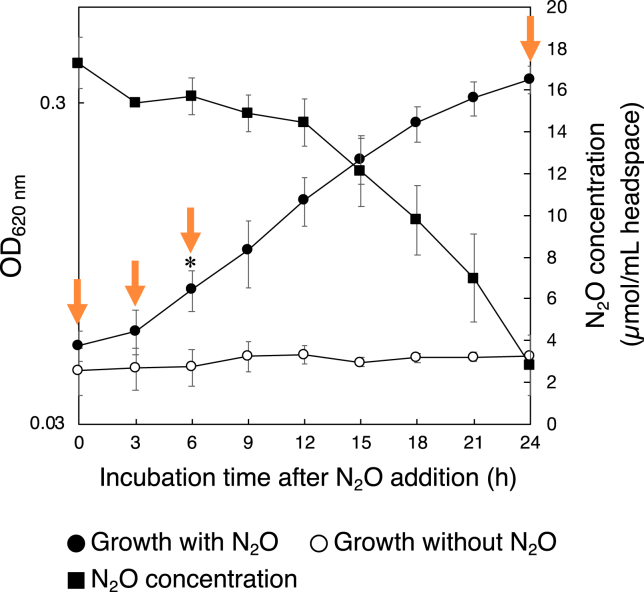
Growth and N_2_O consumption of HRV44^T^ after the addition of N_2_O Growth with N_2_O and N_2_O concentration in the headspace were measured in triplicate, and growth without N_2_O was measured in duplicate. (mean ± standard deviation). Orange arrows represent sampling points for total RNA extraction. Asterisk (∗) represents the first time that detected significant cell growth compared to 0 h (*p* < 0.05).

Strain HRV44^T^ showed a low cell density (avg. OD_620 nm_ = 0.05) after 24 h-cultivation at 55°C in media supplemented with H_2_ (66.6%, *v*/*v*), O_2_ (0.1%, *v*/*v*), and CO_2_ (33.3%, *v*/*v*) to serve as an electron donor, electron acceptor, and carbon source in a headspace of each vial, respectively (Figure S1A). The cell density significantly increased from 6 h (avg. OD_620 nm_ = 0.08) after the addition of N_2_O relative to 0 h at the time of N_2_O addition (*p* < 0.05), with visible bubbles at the gas-liquid interphase (Figures 1 and S1A). The OD_620 nm_ value reached more than 0.30 at 24 h after the addition of N_2_O. N_2_O concentration in the headspace started to decrease 3 h after the addition of N_2_O, and N_2_O consumption during 24 h-cultivation was 14.5 ± 0.85 μmol/mL headspace. No significant growth was detected under the N_2_O-free treatment. Total RNA was extracted before the addition of N_2_O at 0 h (N_2_O-free treatment) and at 3, 6, and 24 h after the addition of N_2_O (N_2_O-added treatment) in triplicate. Since the strain can utilize nitrate (NO_3_^−^) and elemental sulfur (S^0^) as alternative electron acceptors to N_2_O,^14^ HRV44^T^ was also grown in MMJHS medium that contains NO_3_^−^, thiosulfate (S_2_O_3_^2−^), and S^0^ in the liquid phase as previously described.^22^ The cell density reached an average of 0.16 of OD_620 nm_ at 24 h, and OD_620 nm_ values did not fluctuate after 24 h (Figures S1C and S1D). No N_2_O accumulation was detected in the headspace of cultures grown in MMJHS medium over 48 h (Figure S1D). An average 51 μg of total RNA was also extracted after 24 h in MMJHS treatment for comparison to cells grown in N_2_O-free (0 h) and N_2_O-added (3, 6, and 24 h) treatments.

### Transcriptome analyses

**Figure 2:**
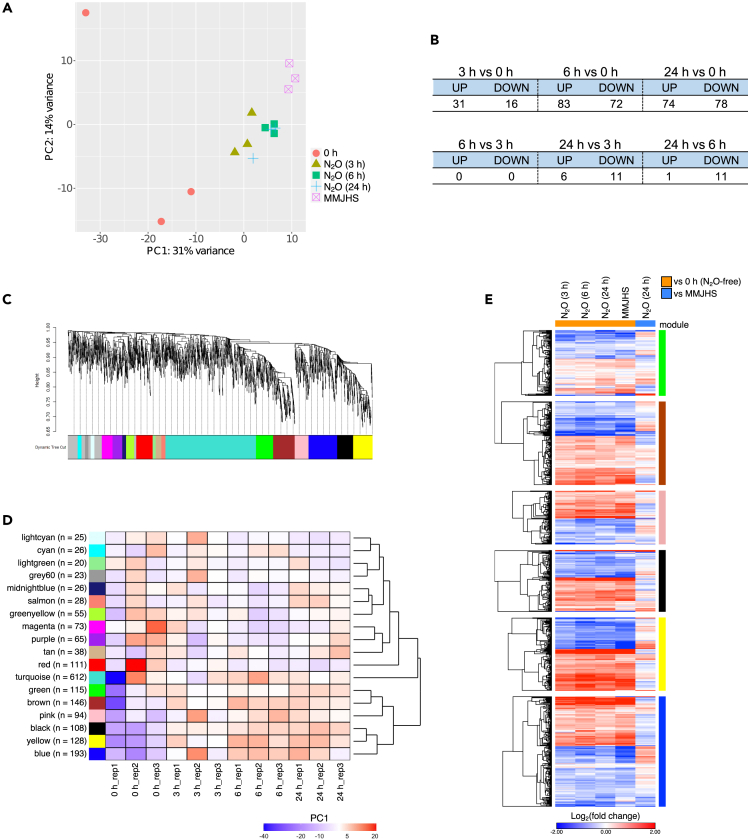
Transcriptome analyses of HRV44^T^ chromosome (A) PCA plot of time course data without and with N_2_O (0, 3, 6, 24 h, and MMJHS). (B) Time course DEG extraction. The list represents the number of the DEGs between N_2_O-added and N_2_O-free treatments (upper) and among N_2_O-added treatments (lower). (C) Hierarchical cluster tree of 1,886 genes (92%) showing 18 modules of co-expressed genes extracted by WGCNA. The lower panel shows modules in designated colors. (D) Dendrogram heatmap constructed by PC1 of each color module. The color key represents PC1 values. (E) Heatmap of log_2_FC values of genes clustered in the six WGCNA modules (the green, brown, pink, black, yellow, and blue modules).

We used an Oxford Nanopore PCR-based RNA-seq method to identify genes that were up- and downregulated in response to N_2_O reduction in HRV44^T^. More specifically, we evaluated differences in transcript expression levels in N_2_O-free (0 h) and N_2_O-added (3, 6, and 24 h) treatments, and in MMJHS (24 h) treatment amended with electron acceptors other than N_2_O. We excluded expression analyses for genes on a plasmid, as >78% of the genes were annotated as hypothetical with unknown functions. Principal-component analysis (PCA) revealed that biotriplicates from N_2_O-added (3, 6, and 24 h) and MMJHS treatments (24 h) yielded transcriptomic datasets that formed a cohesive cluster along axis PC1 and, to some extent, along axis PC2, respectively (Figure 2A). That said, there was a large variation in the transcriptomic data, however, resulting from N_2_O-free (0 h) triplicates. The DESeq2 analysis of the same time course data (0, 3, 6, and 24 h) identified a total of 220 differential expression genes (DEGs) with |log_2_(fold change)| ≥ 1 and a false discovery rate (FDR) < 0.05, accounting for 10.7% of the genes on the chromosome. Compared to the N_2_O-free treatment (0 h), 47, 155, and 152 DEGs were identified at 3, 6, and 24 h after the addition of N_2_O, respectively (Figure 2B). No DEGs were identified between 3 h and 6 h samples. A hierarchical clustering heatmap constructed using expression data from all 220 DEGs produced two major clusters, representing N_2_O-free and N_2_O-added treatments (Figure S2). Weighted gene co-expression network analysis (WGCNA) was also performed on all protein-coding sequences (CDSs) using only the N_2_O-added time course data to reduce the effects on DEG detection caused by large data variation in 0 h triplicates. A total of 1,886 genes (92%) were clustered into 18 modules (Figure 2C). A hierarchical clustering heatmap constructed using the first principal component (PC1) of each colored module illustrated that the green (*n* = 115), brown (*n* = 146), pink (*n* = 94), black (*n* = 108), yellow (*n* = 128), and blue (*n* = 193) modules formed a conserved cluster (Figure 2D). Most of the PC1 values for these six modules changed from negative to positive values after N_2_O was added (0 h versus 3, 6, and 24 h), indicating that genes in all six modules were differentially expressed after the addition of N_2_O. Similar fluctuation trends were observed in the MMJHS treatment compared to the N_2_O-free (0 h) treatment, with different magnitudes of log_2_FC values (Figure 2E). Of the 220 DEGs from the N_2_O experiment, 183 DEGs were included in the six WGCNA modules: 8 in green, 29 in brown, 8 in pink, 31 in black, 72 in yellow, and 35 in blue modules.

**Figure 3:**
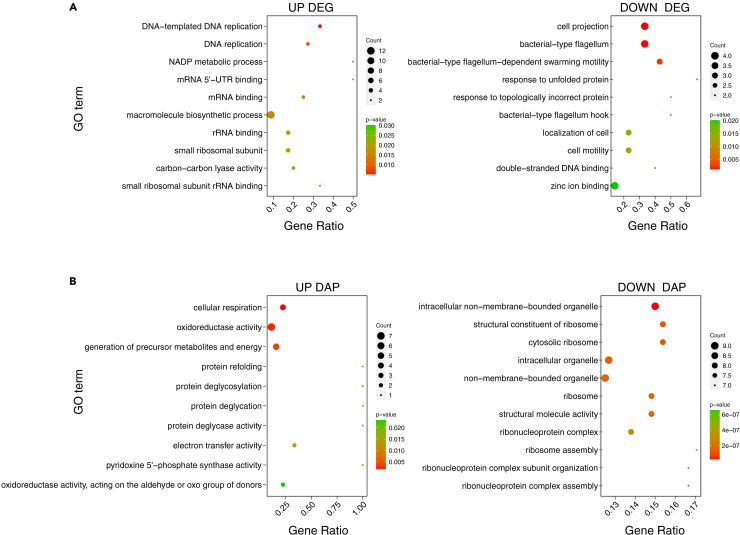
GO enrichment analysis of the DEGs and DAPs (A) The top 10 enriched GO terms in up- and downregulated DEGs filtered by WGCNA. (B) The top 10 enriched GO terms in increased and decreased DAPs.

Gene Ontology (GO) enrichment analysis of the WGCNA-fileted 183 DEGs, assigned to the six WGCNA modules mentioned previously, revealed that 51 (biological process [BP], 21; molecular function [MF], 24; cellular component [CC], 6) and 36 (BP, 15; MF, 15; CC, 6) GO terms were significantly enriched (over-represented *p* value <0.05) in the filtered up- and downregulated DEGs, respectively. Upregulated DEGs were enriched in DNA replication-related GO terms (GO: 0006260 and 0006261) (Figure 3A). Downregulated DEGs were enriched in cell projection (GO: 0042995), bacterial-type flagellum (GO: 0009288), and bacterial-type flagellum-dependent swarming motility (GO: 0071978) GO terms.

A comparison of N_2_O-added (24 h) and MMJHS (24 h) treatments found that 21 genes were significantly upregulated and 18 genes were downregulated in the N_2_O-added (24 h) treatment relative to the MMJHS (24 h) treatment (Table S1). The top enriched GO terms of the integrated DEGs included homeostatic process (GO: 0042592), cellular homeostasis (GO: 0019725), and oxidoreductase activity, acting on a sulfur group of donors (GO: 0016667) (Figure S3).

### Proteome analyses

We performed mass spectrometry-based proteome analyses on cells cultured in N_2_O-free (0 h, *n* = 3) and N_2_O-added (3, 6, and 24 h after the addition of N_2_O, each *n* = 1) treatments to elucidate the response of HRV44^T^ to N_2_O at the protein level. QPROT^23^ analysis identified 48 differential abundant proteins (DAPs) that were significantly increased and 30 that were significantly decreased in abundance using a threshold of |log_2_(fold change)| ≥ 0.5 and |z-statistic| ≥ 2 (Figure S4; Table S2). GO enrichment analysis of the DAPs revealed that 43 (BP, 14; MF, 24; CC, 5) and 118 (BP, 70; MF, 30; CC, 18) GO terms were significantly enriched (over-represented *p* value <0.05) in increased and decreased DAPs, respectively. The top 10 enriched GO terms with increased DAPs in N_2_O-added versus N_2_O-free treatments included cellular respiration (GO: 0045333), oxidoreductase activity (GO: 0016491), and generation of precursor metabolites and energy (GO: 0006091) (Figure 3B). Those with decreased DAPs included intracellular non-membrane-bounded organelle (GO: 0043232), structural constituent of ribosome (GO: 0003735), and cytosolic ribosome (GO: 0022626).

### Comparison of the DEGs and DAPs

**Figure 4:**
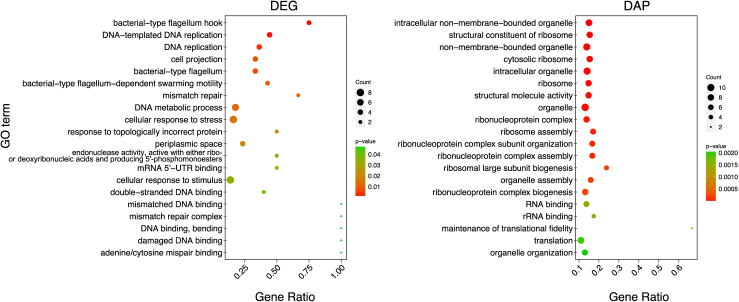
The top 20 enriched GO terms of the integrated DEGs and DAPs

To compare transcriptome and proteome results, DEG analysis was re-performed with the sample group comparison used for the proteomics: N_2_O-free (0 h, *n* = 3) and N_2_O-added data (3, 6, and 24 h, total *n* = 9). DEGs were then filtered based on the six modules identified by WGCNA, as for the time course DEG analysis to reduce effects on DEG detection caused by large data variation in 0 h samples. The low correlations (0.21 ≤ R ≤ 0.33) were observed between transcriptome and proteome results in this study (Table S3), and there was little correspondence in differential mRNA and protein abundances between the DEGs and DAPs. One upregulated DEG and two downregulated DEGs corresponded to an increased DAP, ribonucleotide reductase of class Ⅲ (anaerobic) large subunit (WP_187646885.1), and decreased DAPs, thiol peroxidase Tpx-type (WP_187647451.1) and cytochrome *c* oxidase (*cbb*_*3*_-type) subunit CcoO (WP_187647411.1), respectively (Figure S5). In contrast, one upregulated DEG was identified as the decreased DAP, RNA-binding protein (WP_187647811.1), and two downregulated DEGs were identified as increased DAPs, N-acetyltransferase (WP_187648738.1) and nitric-oxide reductase subunit C (WP_187648292.1). Integrated GO enrichment analysis showed that the top 20 GO terms include bacterial-type flagellum hook (GO: 0009424) and DNA-replication-related (GO: 0006260 and 0006261) for the DEGs and intracellular non-membrane-bounded organelle (GO: 0043232) and structural constituent of ribosome (GO: 0003735) for the DAPs (Figure 4).

### Transcriptomic and proteomic profiling of denitrification genes/proteins

**Figure 5:**
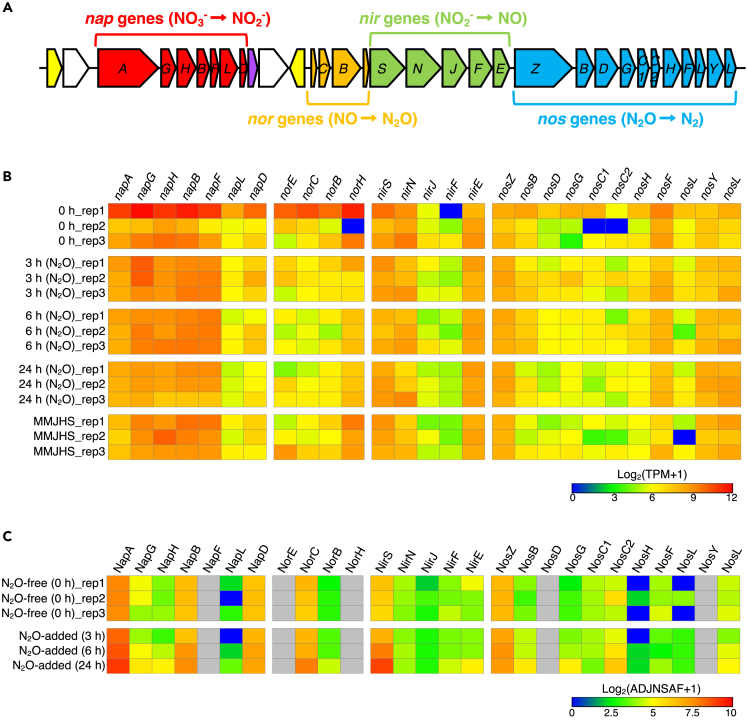
Schematic structure of denitrification genes in HRV44^T^ and their gene and protein expression levels (A) Red, periplasmic nitrate reductase genes (*nap*); orange, nitric oxide reductase genes (*nor*); light green, *cd*_*1*_ nitrite reductase genes (*nir*); light blue, clade Ⅱ nitrous oxide reductase genes (*nos*); yellow, genes encoding Crp/Fnr superfamily transcriptional regulators; purple, PAS domain-containing protein; white, hypothetical protein genes. (B) Heatmap of log_2_(TPM+1) values represents the expression level of the denitrification genes. (C) Heatmap of log_2_(ADJNSAF+1) values represents the expression level of the denitrification proteins. Gray, non-detected proteins among all samples. The heatmaps were generated by the Morpheus web tool (https://software.broadinstitute.org/morpheus/).

In addition to *nos* genes, HRV44^T^ possesses all additional genes needed for complete denitrification, including periplasmic nitrate reductase genes (*nap*, NO_3_^−^ → NO_2_^−^), *cd*_*1*_ nitrite reductase genes (*nir*, NO_2_^−^ → NO), and nitric oxide reductase genes (*nor*, NO → N_2_O) (Figure 5A). The mRNA and protein abundances of those genes were normalized to transcripts per million (TPM) and adjusted normalized spectral abundance factor values (ADJNSAF), respectively. Most of the denitrification genes were constituently expressed, including in the absence of any nitrogen oxides as electron acceptors. Three of the key enzyme genes required for denitrification (*napA*, *nirS*, and *nosZ*) had relatively high TPM values in all samples (Figure 5B). In one replicate from the 0 h time point, *nap* and *nor* genes were characterized by higher log_2_(TPM+1) values compared to the other samples, highlighting the biological variability between cultures. This result is comparable with the proteomics result showing the relatively high log_2_(ADJNSAF+1) values of the three key enzymes (NapA, NirS, and NosZ) (Figure 5C).

### Transcriptomic and proteomic profiling of genes/proteins involved in electron transport pathway to the respiration systems

**Figure 6:**
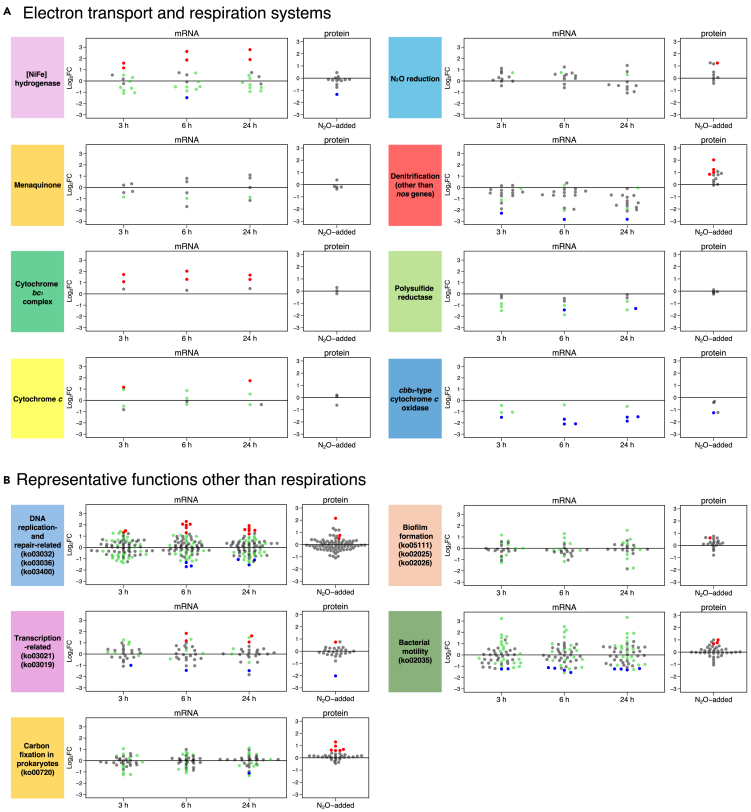
Expression fluctuations of mRNA and proteins in pathways involved in N_2_O reduction Log_2_FC values of mRNAs and proteins related to respiration (A) and other representative functions (B) between N_2_O-free and N_2_O-added treatments. Red and blue points represent the up- and downregulated filtered DEG and DAP, respectively, and green points in mRNA represent “non-DEG, but included in the six WGCNA modules (the green, brown, pink, black, yellow, and blue modules).”

HRV44^T^ possesses denitrification respiration systems, a microaerobic respiration system (*cbb*_*3*_-type cytochrome *c* oxidase), and a sulfur reduction system (polysulfide reductase) (Figure S6). It has been demonstrated that HRV44^T^ is able to use elemental sulfur and molecular oxygen (up to 1%, *v*/*v*) as sole electron acceptors in addition to N_2_O.^14^ The electron donation is attributed solely to the oxidation of H_2_ (H_2_ → 2H^+^ + 2e^−^) in HRV44^T^ catalyzed by H_2_-uptake [NiFe] hydrogenase.^24^ The expression fluctuations of the related genes were shown at mRNA and protein levels between N_2_O-free and N_2_O-added treatments (Figure 6A). Transcriptome analysis revealed that one subunit gene (*hydB*) expression was significantly downregulated 6 h after the addition of N_2_O, whereas expressions of two genes encoding [NiFe] hydrogenase maturation proteins (*hypDE*) were significantly upregulated 3 h after the addition of N_2_O. Hydrogenase maturation protease HybP was a decreased DAP in the N_2_O-added treatment relative to the N_2_O-free treatment. Gene and protein expression levels involved in menaquinone synthesis did not fluctuate significantly. Genes encoding cytochrome *bc*_*1*_ complex subunits (*petAC*) were consistently detected as upregulated DEGs at 3 h after the addition of N_2_O. KEGG pathway and BLAST analyses identified four genes encoding cytochrome *c* (three cytochrome *c*_*553*_ and one cytochrome *c*_*552*_). All three cytochrome *c*_*553*_ genes were included in the six WGCNA modules mentioned previously (Figure 2D), and the expression of one of them (*cccA*) was significantly upregulated in the N_2_O-added treatment. The observed fluctuations in mRNA and protein expressions related to electron transport in respiration did not exhibit consistent trends. Of the 11 *nos* genes, only the *nosZ* gene was included in the six WGCNA modules (the blue module) with >0.7 log_2_FC at FDR ≤0.1 at 3 h and 6 h after the addition of N_2_O compared to the N_2_O-free treatment (0 h), although it was not detected as a DEG (Figure S7). This trend for *nosZ* obtained from transcriptome analysis is consistent with the proteome analysis, for which NosZ was not detected as a DAP (log_2_FC = 0.498 and z-statistic = 3.73). There were large discrepancies in log_2_FC values between transcriptomic and proteomic data for denitrification genes other than *nos* (*nap*, *nir*, and *nor*). Although most of the log_2_FC values for these genes calculated from RNA-seq data were negative, the key enzymes and their components (NapAB, NirS, and NorC) were increased DAPs in the N_2_O-added treatment (Table S2A). In addition, the expression levels of the *nap*, *nir*, *nor*, and *nos* genes did not show statistically significant differences between the N_2_O-added (24 h) and MMJHS treatments (Table S1). Gene expression for components of polysulfide reductase (*psrA*_*1*_*B*_*1*_*CD*) did not significantly fluctuate, with the exception of a downregulated chaperone (*psrE*). One subunit gene of *cbb*_*3*_-type cytochrome *c* oxidase (*ccoO*) was detected as a downregulated DEG at 3 h after the addition of N_2_O, while other subunit genes (*ccoPQ*) exhibited significantly downregulated expressions at 6 h after the addition of N_2_O. Significant downregulation in CcoO expression was also observed in the proteome (Table S2B).

### Transcriptomic and proteomic profiling of genes/proteins involved in functions other than respirations

We further examined expression fluctuations of the genes and proteins important for DNA replication, transcription, carbon fixation, biofilm formation, and bacterial motility (Figure 6B). Among the DNA replication- and repair-related genes (DNA replication protein, ko03032; chromosome and associated protein, ko03036; and DNA repair and recombination protein, ko03400), two genes (encoding single-strand DNA-binding protein and ribonuclease HII) were consistently upregulated at 3 h after the addition of N_2_O. Four additional genes (encoding rod shape-determining protein RodA, ATP-dependent DNA helicase Rep, helicase PriA, and DNA polymerase Ⅲ subunits gamma and tau) were also significantly upregulated. Three increased DAPs related to these functions (DNA-binding protein HU, DNA translocase FtsK, and DNA polymerase X family/PHP domain protein) were also detected (Table S2). Among the genes involved in transcription (transcription machinery, ko03021 and mRNA biogenesis, ko03019), a constant downregulated expression of a gene encoding transcription termination factor Rho was detected in the N_2_O-added treatment. Whereas, upregulated expressions of genes encoding transcription elongation factor GreA and 6-phosphofructokinase were detected at 6 h and 24 h after the addition of N_2_O, respectively. Abundances of transcription termination protein NusB and ATP-dependent RNA helicase DeaD were significantly increased and decreased at the protein level, respectively. The expression of prokaryotic carbon fixation-related (ko00720) genes did not significantly fluctuate during the N_2_O-added treatments, with one exception of a downregulated gene encoding ATP citrate synthase alpha chain. At the protein level, we detected six related proteins with increased abundance (pyruvate:ferredoxin oxidoreductase delta subunit, phosphoenolpyruvate synthase, malate dehydrogenase, heterodisulfide reductase subunit B-like protein, biotin carboxylase of acetyl-CoA carboxylase, and acetyl-CoA synthetase). Furthermore, genes associated with biofilm formation were evaluated based on the KEGG pathway (biofilm formation of *Vibrio cholerae*, ko05111; of *Pseudomonas aeruginosa*, ko02025; and of *Escherichia coli*, ko02026) as HRV44^T^ cells were more active in forming pellicle-like structures under N_2_O-added treatments relative to N_2_O-free and MMJHS treatments (Figures S1B and S1C). Transcriptome analysis did not detect any significant fluctuation of the relevant gene expressions; however, proteomics identified one increased DAP, mannose-1-phosphate guanylyltransferase/mannose-6-phosphate isomerase (AlgA), involved in biofilm formation of *P. aeruginosa* (ko02025). With regard to bacterial motility (ko02035), expressions of the five flagellar component genes were downregulated (*flgBDEK* and *fliG*), while abundances of chemotaxis proteins CheBY and flagellin protein FlaA showed significant increases at the protein level.

### Prediction of transcriptional units of the denitrification genes and binding sites of putative transcriptional regulators

HRV44^T^ possesses two genes that encode Crp/Fnr superfamily proteins (NIL_RS03760 and NIL_RS03815) upstream of the *nap* and *nor* gene clusters, respectively (Figure 5A). NIL_RS03760 was extracted as a downregulated DEG at 6 h after the addition of N_2_O and included in the yellow module, which showed a shift in expression before and after the addition of N_2_O (Table S4). Values of amino acid identities (AAI) of NIL_RS03760 and NIL_RS03815 against NssA, NssB, and NssC, Crp/Fnr superfamily proteins of *W*. *succinogenes* were 44.8%, 29.2%, and 26.1% and 33.3%, 50.0%, and 28.2%, respectively (Table S5).

RNA-seq coverage indicates that the number of mapped reads was much lower in non-coding regions of the upstream of *nap* and *nor* gene clusters, *nosZ*, and *nosB* genes (Figure S8A). We searched the binding sites for Crp/Fnr-type transcriptional regulators by allowing for a two-bp mismatch compared to the consensus sequence (TTGA-N_6_-TCAA).^18^ We identified five, three, four, and one binding sequences upstream of the predicted transcription start sites (TSS) of *napA*, *norE*, *nosZ*, and *nosB* genes, respectively (Figure S8B). These included one exact match upstream of each *napA*, *norE*, and *nosB* gene. Distances from the initial base of the exact Crp/Fnr superfamily protein binding sequence to TSS were 49, 48, and 48 bases upstream of *napA*, *norE*, and *nosB* genes, respectively. The binding sequences overlapping the predicted TSS were identified upstream of the *nosZ* and accessory *nos* genes. Distances between each TSS and predicted ribosome binding site (RBS) were 37, 37, 15, and 15 bases upstream of *napA*, *norE*, *nosZ*, and *nosB* genes, respectively.

### Correlation and causal relationship between the denitrification genes and Crp/Fnr superfamily protein genes

Nss proteins belonging to the Crp/Fnr superfamily have been reported to mediate the upregulation of denitrification proteins responding to nitrogen compounds in *W. succinogenes*.^18^ We examined to see if there was a correlation between the expression of genes encoding Crp/Fnr superfamily proteins and denitrification genes. Pearson correlation coefficient (PCC) calculated by time course RNA-seq data normalized with counts per million (CPM) showed that NIL_RS03760 expression had relatively high positive correlations with several *nap*, *nor*, and *nir* gene clusters (R ≥ 0.5), while NIL_RS03815 was not correlated with the same genes (R ≤ 0.2) (Figure S9). In contrast, *nosZ* expression had strong negative correlations with both NIL_RS03760 (R = −0.67, *p* < 0.05) and NIL_RS03815 (R = −0.65, *p* < 0.05).

We also conducted the path analysis using time course RNA-seq data in order to confirm whether the two genes encoding Crp/Fnr superfamily proteins (NIL_RS03760 and NIL_RS03815) contributed to the downregulation of the *nosZ* gene. The analysis found a significantly negative effect of NIL_RS03760 on *nosZ* (*p* < 0.05), while the effect of NIL_RS03815 on *nosZ* was not significant (Figure S10). In the model with the presence of N_2_O as an exogenous variable, N_2_O had a significant effect on both NIL_RS03760 and NIL_RS03815 (Figure S10A). When accounting for the duration of the exposure to N_2_O, as an exogenous variable, N_2_O had no significant effect on the gene expressions (Figure S10B). The first model resulted in better model fit scores, indicating that the presence or absence of N_2_O can better explain the data compared to the duration of N_2_O exposure. Although the direct effect of N_2_O on *nosZ* was not significant in both models, the first model found an indirect effect mediated by NIL_RS03760 (coefficient = 0.69, *p* < 0.05), indicating the suppression of NIL_RS03760 by N_2_O and by increased *nosZ* expression.

## Discussion

### Transcriptome and proteome profiling

Our experiment was designed to characterize shifts in strain HRV44^T^ gene expression in response to N_2_O. We showed that HRV44^T^ exhibited a transcriptional response to the addition of N_2_O within at least 3 h. Statistically significant changes in gene expression were identified in 10% of the chromosome, which were maintained throughout the course of the 24-h experiment. This suggests that HRV44^T^ response to N_2_O-rich environment is rapid and persistent. GO enrichment analysis of the transcriptomics and proteomics represented that the functions related to DNA replication, respiration, and energy generation were activated under the N_2_O-added treatment. These indicate that HRV44^T^ increased its cellular activity under the N_2_O-added condition relative to the N_2_O-free condition.

N_2_O was not a crucial inducer of the denitrification genes (*nap*, *nir*, *nor*, and *nos*) in HRV44^T^. The induction of those genes may be particularly mediated by anaerobic stimuli that modulate O_2_-responsive transcriptional regulators (discussed in the following text) or by regulation by sigma factor 70, which is responsible for regulating the transcription of most genes expressed during exponential cell growth.^25^ HRV44^T^ had the highest growth with N_2_O-respiration, and none of the other terminal electron acceptors provided comparable cell densities for HRV44^T^ as N_2_O-respiration (data not shown). Because of this distinctive adaptation and finding, it was difficult to select a control treatment for comparing gene expression during cell growth with N_2_O. Although we did not perform time course transcriptome analysis on the MMJHS treatment, it should be noted that the shift in gene expression detected in the N_2_O-added treatment was not specific and also occurred when MMJHS medium was used, which contained S^0^ and NO_3_^−^ as alternative electron acceptors. Indeed, no DEGs for sulfur-reduction or nitrate-reduction were detected in the N_2_O-added treatment compared to the MMJHS treatment, suggesting that HRV44^T^ proliferates at high efficiency by possessing a regulation system that is independent of the electron acceptor type. Further analysis for evaluating time course gene and protein expressions on each electron acceptor is needed to better understand transcript and protein expression responses to different types of electron acceptors.

### Putative regulation mechanism of denitrification gene expressions: Negative regulation of *nosZ* gene expression mediated by Crp/Fnr superfamily protein

Crp/Fnr superfamily proteins are involved in responses to a variety of signals, such as oxygen, carbon monoxide, temperature, nitric oxide, and oxidative and nitrosative stress.^26^ Our RNA-seq analyses demonstrate that the expression of a Crp/Fnr superfamily protein (NIL_RS03760) located upstream of the *nap* cluster had a significantly negative causal relation with that of the *nosZ* gene. This is the study suggesting a negative regulation of Crp/Fnr superfamily regulators on expression of *nosZ*. HRV44^T^ has two genes encoding Crp/Fnr superfamily proteins (NIL_RS03760 and NIL_RS03815) that are 44.8% and 50.0% identical with NssA and NssB in *W. succinogenes*, respectively. N_2_O has been reported as a possible signal of Crp/Fnr superfamily proteins for *W*. *succinogenes*, mediating the upregulation of NapA, cytochrome *c* nitrite reductase (NrfA), and NosZ.^18^ NssA and NssB, possessing a Dnr-type C-terminal DNA-binding domain, are known as homologues of the nitrosative stress sensing regulator NssR in *Campylobacter jejuni*.^27^ The Dnr system is involved in an NO sensory pathway and expressed in the absence of oxygen or lowered oxygen tension and in the simultaneous presence of nitrogen oxide.^26^ We found binding sequences of a Dnr-type regulator belonging to Crp/Fnr superfamily protein upstream of four transcriptional units of denitrification genes, (1) *nap* genes, (2) *nor* and *nir* genes, (3) *nosZ* gene, and (4) accessory *nos* genes, inferred by visualized mapping data (Figure S8). These results suggest that the denitrification genes are under the control of transcription factors belonging to this regulatory family in HRV44^T^, resulting in the stable expression of the denitrification genes and proteins before and after the addition of N_2_O under anaerobic conditions. Further explorations, such as conducting transcriptomics at different O_2_ levels, detection of promoter sequences (−10 and −35 elements), and construction of mutants in these putative transcription regulators, are needed in order to identify the exact function of the transcriptional regulators and the presence of an N_2_O-sensing mechanism.

### Hydrogen oxidation and electron transfer pathway accompanied with N_2_O-reducing activity of HRV44^T^

Hydrogen serves as an electron donor for HRV44^T^ during N_2_O respiration.^14^ Hydrogen oxidation is known to be catalyzed by the respiratory H_2_-uptake [NiFe] hydrogenase. The [NiFe] hydrogenase subunit proteins were detected at a relatively high level under both N_2_O-free and N_2_O-added treatments, and their abundances did not significantly fluctuate. Prediction of bacterial promoters indicated that there are one (Fur-dependent) and two (sigma70 and Fnr-dependent) promoters upstream of *hypB* and *hypE* genes, which are indispensable for maturation of [NiFe] hydrogenase, respectively (data not shown), suggesting a different transcriptional regulation in *hyp* genes in strain HRV44^T^ given by a variety of stimuli. Since the *hyp* operon expression is upregulated in anaerobic conditions in *E. coli*,^28^ full depletion of O_2_ or abundant N_2_O may cause further anaerobic stimuli.

Two electron-transfer pathways have been proposed to be related to bacterial N_2_O reduction: from cytochrome *bc*_*1*_ complex to NosZ via cytochrome *c* (common in both clade Ⅰ and Ⅱ N_2_O-reducing bacteria) and via NosGH, NosBC2 complexes, and NosC1 (unique in clade Ⅱ N_2_O-reducing bacteria).^29^^,^^30^ In this study, significant upregulation was detected in *cccA* gene encoding cytochrome *c*_*553*_ and in subunit genes (*petAC*) of cytochrome *bc*_*1*_ complex. In *P. denitrificans*, a clade I-type microorganism, a higher level of cytochrome *c*_*553*_ was detected in cells grown N_2_O-anaerobically than cells grown high-aerobically or NO_3_^−^-anaerobically.^31^ Our results present that HRV44^T^ may prefer the electron transport to NosZ via cytochrome *c*_*553*_ rather than via electron-transporting accessory Nos proteins. In addition to transcriptomic data, proteins related to electron transport were included in the top 10 GO term (electron transfer activity) for increased DAPs and detected as two DAPs, i.e., electron transfer flavoprotein (ETF) alpha subunit and electron transfer flavoprotein-ubiquinone oxidoreductase (ETF-QO), which are related to electron transfer to the quinone pool.^32^ The relation between electron transport and N_2_O reduction has been mentioned in previous studies. For instance, the N_2_O-reducing activity of *Bradyrhizobium diazoefficiens* with clade Ⅰ type *nosZ* with deletion of *cycA* gene encoding soluble cytochrome *c*_*550*_, which functions as an intermediate electron donor, was decreased by about 65% compared to wild-type cells.^33^ Taken together, we hypothesize that the electron-transport activity is one of the crucial factors underlying the N_2_O-reducing ability in HRV44^T^, which is enhanced at the transcription level under N_2_O-added treatments. This could occur within 3 h in response to N_2_O, and such a mechanism may help HRV44^T^ to grow at high efficiency and exhibit high N_2_O-reducing activity at a bulk level. Denitrification proteins, *cbb*_*3*_-type cytochrome *c* oxidase, and polysulfide reductase, as well as Nos proteins, were detected in both N_2_O-free and N_2_O-added treatments, implying that there is an electron competition among these proteins and a preference for electron transport pathway among respiration systems in HRV44^T^ though we could not evaluate the electron flow into NosZ.

### Biofilm formation and motility regulation associated with N_2_O reduction

Pellicle is known as a floating biofilm formed at the air-liquid interface in static culture conditions. Although transcriptomics did not detect the significant expression changes of the biofilm formation-related genes, the increased abundance of AlgA in N_2_O-added treatments suggested that the pellicle-like structure is likely to contain alginate. Inhibition of motility of planktonic microbes is required for their biofilm formation,^34^ and the enrichment of GO terms related to motility in downregulated DEGs confirms that motility was likely decreased after the addition of N_2_O (Figure 3A). In addition, genes related to twitching motility (*pilT*) and regulation of flagellar operon expression (*fliA*), as well as flagellar formation, were significantly downregulated (Table S4). PilT is involved in pilus retraction required for pilus-mediated twitching motility.^35^ The expression of genes involved in flagellar formation is regulated by three cascades: class 1, 2, and 3 in pathogenic species within “*Campylobacteria*”, and RNA polymerase sigma factor (σ^28^), encoded by *fliA* gene, is classified into class 2.^36^ FliA regulates the expressions of class 3 genes that contribute to the formation of the major flagellin and minor filament proteins.^36^ Taken together, our results imply that both swimming and twitching motilities were inhibited directly or indirectly in response to N_2_O. This may be an adaptative strategy in which cells decrease motility and create a suitable habitat at the air-liquid surface to access the electron donors and acceptors.

### Conclusion

This study provides a framework for studying time series transcriptome analysis of deep-sea hydrothermal vent microorganisms with Nanopore PCR-based RNA-seq, which requires only 1 ng of rRNA-depleted poly(A) mRNA, making it useful for transcriptome analysis of low-growth bacteria, from which RNA extraction is difficult. Our findings demonstrate the utility of Nanopore PCR-based RNA-seq for difficult-to-culture microorganisms and highlight their strategy for adaptation to deep-sea hydrothermal environments and the presence of N_2_O. N_2_O is, therefore, not a critical inducer of denitrification gene expression in HRV44^T^, and those genes and proteins are expressed under anaerobic conditions even in the absence of nitrogen oxides as electron acceptors. This feature may contribute to efficient energy metabolisms in deep-sea hydrothermal environments where electron acceptors are occasionally depleted in anaerobic environments. Our transcriptome and proteome analyses suggest that the number of electrons transported to NosZ is regulated at the transcription level, depending on the external environment, such as the amount of N_2_O. This mechanism likely contributes to high-efficiency growth via N_2_O respiration, and consequently HRV44^T^ can show high N_2_O-reducing activity at a bulk level. Gene expression comparisons between N_2_O-added and MMJHS treatments, however, revealed that responses were not specific to the N_2_O-added treatment and increased electron-transport efficiency may depend on the existence of the available electron acceptors and on cellular activities. HRV44^T^ can simultaneously reduce N_2_O and CO_2_ under the unique conditions of high temperature (45°C–60°C) and acidic (pH 5.4–6.4) conditions,^14^ making it a particular bioresource that can contribute to mitigating N_2_O emissions.^37^ Our finding that Crp/Fnr superfamily regulators negatively regulate the expression of *nosZ* in HRV44^T^ extends the understanding of regulatory mechanisms of gene expression in clade II type N_2_O-reducers and may help increase their ability of N_2_O reduction.

### Limitations of the study

Although all cultivations with N_2_O were conducted under the condition of 33% (*v*/*v*) N_2_O in the headspace, we were unable to evaluate the intracellular concentrations of nitrogen compounds (e.g., NO_3_^−^, NO_2_^−^, NO, and N_2_O) in addition to those in liquid phases.

A limitation in transcriptomics of this study is the lower throughput of Nanopore-based RNA-seq compared to Illumina RNA-seq, e.g., total raw counts averaged 162,061 and 8,852,252 for Nanopore PCR-based and Illumina RNA-seq, respectively, though positive Pearson correlation coefficients were observed between their normalized data (Table S6). A limitation in proteomics of this work is the limited sample size. A clearer link between the transcriptomic and proteomic datasets might be observed if we had sampled more time points.

## Resource availability

### Lead contact

Further information and requests for resources should be directed to and will be fulfilled by the lead contact, Sayaka Mino (sayaka.mino@fish.hokudai.ac.jp).

### Materials availability

This study did not generate new unique reagents.

### Data and code availability

• Data: the raw RNA-seq datasets presented in this study (fastq files before quality control) can be found in DDBJ Sequence Read Archive (DRA, https://www.ddbj.nig.ac.jp/) and are available under BioProject accession number PRJDB15435 (Run: DRR451265-DRR451279 and DRR571757-DRR571759). The mass spectrometry proteomics dataset has been deposited to the ProteomeXchange Consortium via the PRIDE^38^ partner repository with the dataset identifier PXD053214.
• Code: this paper does not report original code.
• All other requests: any additional information required to reanalyze the data reported will be shared by the lead contact upon request.

## Acknowledgments

This work is partially supported by the 10.13039/100007802Institute for Fermentation, Osaka (S.M.), 10.13039/100008965Takahashi Industrial and Economic Research Foundation (T.S.), 10.13039/501100001691JSPS KAKENHI grant numbers 17K15301 (S.M.) and 21K14913 (S.M.), JSPS Overseas Research Fellowships (S.M.), and JSPS Research Fellowships for Young Scientists (J.T.).

## Author contributions

Conceived and designed the experiments: J.T., S.M., and T.S. Performed transcriptomic experiments: J.T. Performed proteomic mass spectrometry experiments and protein data searches: R.M.M., B.L.N., and E.T.-S. Analyzed transcriptomic and proteomic data: J.T., S.M., F.F., N.O., and Y.I. Performed/contributed reagents/material/analysis tools: S.M., F.F., N.O., Y.I., R.M.M., and T.S. Wrote the draft paper: J.T., S.M., and T.S. All authors have read, edited, and approved the final manuscript.

## Declaration of interests

The authors have no competing interests.

## STAR★Methods

### Key resources table

**Table:** 

REAGENT or RESOURCE	SOURCE	IDENTIFIER
**Bacterial and virus strains**		

*Nitrosophilus labii* strain HRV44^T^	Fukushi et al.^14^	JCM 34002

**Chemicals, peptides, and recombinant proteins**		

EDTA・3Na	Dojindo	Cat# 342-01875
trisodium citrate dihydrate	Wako	Cat# 191-01785
ammonium sulfate	Wako	Cat# 019-03435
sulfuric acid	Wako	Cat# 192-04696
TRIzol Reagent	Invitorgen	Cat# 15596026
*E. coli* Poly(A) Polymerase	New England Biolabs	Cat# M0276
Terminator 5′-Phosphate-Dependent Exonuclease	Lucigen	Cat# TER51020
AMPure XP Reagent	Beckman Coulter	Cat# A63880
Deoxynucleotide (dNTP) Solution Mix	New England Biolabs	Cat# N0447S
Maxima H Minus Reverse Transcriptase (200U/μL) with 5×RT Buffer	ThermoFisher	Cat# EP0751
LongAmp Taq 2X Master Mix	New England Biolabs	Cat# M0287
RNase Inhibitor, Murine	New England Biolabs	Cat# M0314
Exonuclease Ⅰ	New England Biolabs	Cat# M0293

**Critical commercial assays**		

RNeasy Min Elute Cleanup Kit	Qiagen	Cat# 74204
NEBNext Poly(A) mRNA Magnetic Isolation Module	New England Biolabs	Cat# E7490
PCR-cDNA Barcoding Kit	Oxford Nanopore Technologies	SQK-PCB109
Flow Cell Priming Kit	Oxford Nanopore Technologies	EXP-FLP002
MinION FlowCell	Oxford Nanopore Technologies	FLO-MIN106D

**Deposited data**		

The raw RNA-Seq datasets presented in this study (fastq files before quality control)	This paper	BioProject, PRJDB15435; DDBJ Sequence Read Archive, DRR451265-DRR451279 and DRR571757-DRR571759
The mass spectrometry proteomics dataset presented in this study	This paper	ProteomeXchange, PXD053214
Strain HRV44^T^ reference genome	Fukushi et al.^14^	NCBI database, GCF_014466985.1

**Software and algorithms**		

MinKNOW (ver. 3.6.0, ver. 21.06.13)	Oxford Nanopore Technologies	https://nanoporetech.com/
Guppy software (ver. 3.4.1; ver. 5.0.16)	Oxford Nanopore Technologies	https://nanoporetech.com/
NanoFilt (ver. 2.6.0)	Coster et al.^39^	https://github.com/wdecoster/nanofilt
minimap2 (ver. 2.17-r941)	Li^40^	https://github.com/lh3/minimap2
samtools (ver. 1.9)	Li et al.^41^	http://www.htslib.org
featureCounts in Rsubreads software (ver. 1.6.2)	Liao et al.^42^	https://bioconductor.org/packages/release/bioc/html/Rsubread.html
fastp (ver. 0.21.1)	Chen et al.^43^	https://github.com/OpenGene/fastp
Bowtie2 (ver. 2.3.4.3)	Langmead and Salzberg^44^	https://bowtie-bio.sourceforge.net/bowtie2/index.shtml
iDEP.951 or 0.96	Ge et al.^45^	http://bioinformatics.sdstate.edu/idep/
DESeq2	Love et al.^46^	https://bioconductor.org/packages/release/bioc/html/DESeq2.html
Morpheus web tool	Broad Institute	https://software.broadinstitute.org/morpheus/
egg-NOG mapper v2	Cantalapiedra et al.^47^	http://eggnog-mapper.embl.de
goseq (ver. 1.50.0)	Young et al.^48^	https://bioconductor.org/packages/release/bioc/html/goseq.html
REVIGO	Supek et al.^49^	http://revigo.irb.hr
Interactive Genomic Viewer (IGV)	Thorvaldsdóttir et al.^50^	https://igv.org/app/
RAST server	Aziz et al.^51^	https://rast.nmpdr.org
in silico MolecularCloning	In silico biology	https://www.insilico-biology.com/index.php
BlastKOALA	Kanehisa et al.^52^	https://www.kegg.jp/blastkoala/
lavaan (ver. 0.6–13)	Rosseel^53^	https://cran.r-project.org/web/packages/lavaan/index.html
Skyline	MacLean et al.^54^	https://skyline.ms/project/home/software/Skyline/begin.view
Comet v. 2022.01 rev.0	Eng et al.^55^^,^^56^	https://uwpr.github.io/Comet/
CRAPome	Mellacheruvu et al.^57^	www.crapome.org
PeptideProphet	Nesvizhskii et al.^58^	https://peptideprophet.sourceforge.net
ProteinProphet	Nesvizhskii et al.^58^	https://proteinprophet.sourceforge.net
Abacus	Fermin et al.^59^	https://abacustpp.sourceforge.net
QPROT	Choi et al.^23^	https://sourceforge.net/projects/qprot/

### Experimental model and study participant details

#### Culture conditions

*Nitrosophilus labii* HRV44^T^ was precultured in 3 mL of N_2_O-minus modified HNN medium^13^ for 24 h at 55°C. The medium contained 0.1% (w/v) NaHCO_3_ per liter of modified MJ synthetic seawater.^60^ Modified MJ synthetic seawater is composed of 25 g NaCl, 4.2 g MgCl_2_・6H_2_O, 3.4 g MgSO_4_・7H_2_O, 0.5g KCl, 0.25 g NH_4_Cl, 0.14 g K_2_HPO_4_, 0.7 g CaCl_2_・2H_2_O, and 10 mL trace mineral solution per liter of distilled water. To prepare the N_2_O-minus modified HNN medium, a concentrated solution of NaHCO_3_ was added before gas purging of 100% CO_2_. The tubes were then tightly sealed with butyl rubber stopper, autoclaved, and pressurized the headspace to 300 kPa with 100% H_2_. Then 0.1% (v/v) of O_2_ was injected into each tube. H_2_ and O_2_ were the sole electron donor and acceptor of the medium, respectively. Ammonium was the only source of nitrogen in the medium. A total of 2 mL of preculture was inoculated to 50 mL glass vials of 20 mL of the same medium, and vials were incubated statically at 55°C for 24 h. Vials were depressurized with 27G needles (TERUMO, Japan), and 46 mL of N_2_O equivalent to the volume of headspace was injected into each vial, which was then pressurized with mixed gas (80% H_2_ + 20% CO_2_) to 300 kPa. No other nitrogen oxide was included in this medium. In addition, strain HRV44^T^ was precultured for 24 h at 55°C in MMJHS medium^22^ that contained nitrate (NO_3_^−^), thiosulfate (S_2_O_3_^2−^), and elemental sulfur (S^0^) in liquid phase (MJ synthetic seawater), and H_2_ (80%) and CO_2_ (20%) in the gas phase. Then, 2 mL of preculture was inoculated to 50 mL glass vials of MMJHS medium, and vials were incubated statically at 55°C for 24 h.

### Method details

#### Measurement of growth and N_2_O consumption

During cultivation after N_2_O treatment, 500 μL of culture medium and headspace gas were collected at each time point (*t* = 0 (before the addition of N_2_O), 3, 6, 9, 12, 15, 18, 21, and 24 h). Then, optical density (OD) and N_2_O concentration in the headspace were measured using TECAN infinite200 (absorbance, 620 nm) and a gas chromatograph (Shimadzu, Japan) with the SHINCARBON ST 50/80 (2 m × 3 mm) column (Shinwa Chemical Industries, Japan), respectively. The cultivation experiment was carried out in triplicate, and statistical significance was checked by a paired *t*-test. In addition, 500 μL of culture medium and headspace gas were collected at each time point (*t* = 0, 12, 24, 36, and 48 h) during cultivation in MMJHS medium, and OD_620 nm_ and N_2_O concentration in the headspace were measured.

#### RNA preparation for nanopore PCR-based RNA-Seq

Collecting bacterial samples for RNA extraction was conducted in N_2_O-free (*t* = 0 h, before the addition of N_2_O, n = 3), N_2_O-added (*t* = 3, 6, and 24 h after the addition of N_2_O, n = 3), and MMJHS (*t* = 24 h, n =3) treatments. 20 mL of culture medium in each vial were transferred into 50 mL plastic centrifuge tubes, and then 10 mL of RNA fixation solution (8.24 g/L EDTA・3Na, 7.36 g/L trisodium citrate dihydrate, and 700 g/L ammonium sulfate in nuclease-free water, pH = 5.2 adjusted by 1 M H_2_SO_4_) was added to each centrifuge tubes. This mixed liquid was centrifuged (16,000×*g*, 20 min, 4°C), and the pellet was dissolved in 1 mL of TRIzol Reagent (Invitrogen, USA) and stored at -80°C. Total RNA was extracted with the following methods. The frozen TRIzol solution in which the bacterial pellets were suspended was thawed at room temperature (RT) for 30 min, mixed by inverting the tube, and allowed to stand at RT for 5 min. Then, 200 μL chloroform was added, mixed by inversion for 15 sec, and allowed to stand at RT for 15 min. After centrifuge (12,000×*g*, 4°C, 15 min), a transparent layer (about 500 μL) was transferred into a new tube, then 1 μL glycogen (5 μg/μL) and 500 μL isopropanol were added to the new tube, mixed by inverting and allowed to stand at RT for 10 min. After centrifuge (12,000×*g*, 4°C, 10 min) and discarding supernatant, the pellet was washed with 1 mL of 75% ethanol and centrifuged (12,000×*g*, 4°C, 5 min). Discarding the supernatant, the pellet was allowed to dry at RT for 30 min. Added 55 μL nuclease-free water, the tube was incubated at 55°C for 10 min and cooled at 4°C for 1 h, followed by the cleanup process using the RNeasy MinElute Cleanup kit (Qiagen, Germany). Total RNA integrity was measured with a spectrophotometer (BioSpectrometer, Eppendorf, Germany). *E. coli* Poly(A) polymerase (New England Biolabs, USA) and Terminator 5’-Phosphate-Dependent Exonuclease (Lucigen, USA) were used to perform polyadenylation and remove rRNA, respectively. Poly(A) mRNA was purified using NEBNext Poly(A) mRNA Magnetic Isolation Module (New England Biolabs, USA). All the above steps were performed according to the protocols provided by the manufacturers, if not mentioned.

#### Library preparation and nanopore PCR-based RNA-Seq

The cDNA library was prepared using the PCR cDNA Barcoding kit (SQK-PCB109) (Oxford Nanopore Technologies, UK). cDNA amplification was performed by 17 PCR cycles (within a range of the protocol provided by Oxford Nanopore Technologies). The cDNA library was loaded into the FlowCell (FLO-MIN106D) on MinION devices (Mk1B or Mk1C) and performed a sequencing run with MinKNOW software (ver. 3.6.0 for time-course data, ver. 21.06.13 for MMJHS data), generating fast5 files. All the above steps were performed according to the protocols provided by the manufacturers, if not mentioned. Sequencing statistics are available in Table S7.

#### RNA preparation for illumina RNA-Seq

To evaluate the potential application of Nanopore PCR-based RNA-Seq, total RNA for Illumina RNA-Seq was also extracted from the treatments with N_2_O (24h) in triplicate by the same method mentioned above. rRNA removal and library preparation were conducted using RiboZero Plus rRNA Depletion Kit and TruSeq stranded mRNA, respectively, at Research Institute for Microbial Disease, Osaka University. RNA-Seq was performed (100 bp, single end) in triplicate with Illumina NovaSeq6000.

#### Pre-processing of nanopore PCR-based RNA-Seq data

The fast5 read files generated from Nanopore PCR-based RNA-seq were basecalled and sorted by barcode with Guppy software (time-course data, accurate model, ver. 3.4.1; MMJHS data, fast model, ver. 5.0.16), generating fastq files. Raw sequence reads were trimmed with NanoFilt^39^ (ver. 2.6.0) to remove reads with an average quality score below 6 or less length than 100 bases. Trimmed reads were mapped to strain HRV44^T^ genome with minimap2^40^ (ver. 2.17-r941), and then generated sam files were sorted and converted to bam files with samtools^41^ (ver. 1.9). The number of reads mapped to CDSs of the HRV44^T^ genome successfully was counted with featureCounts^42^ in Rsubreads software (ver. 1.6.2). High correlations (R^2^ > 0.99) were confirmed between the raw count of the time-course data basecalled by accurate and fast models. The genome sequence and annotation data of HRV44^T^ were retrieved from the NCBI database (GCF_014466985.1). Gene name and annotation were also referred to data from egg-NOG mapper v2^47^ (http://eggnog-mapper.embl.de) and RAST server^51^ (https://rast.nmpdr.org), respectively. The functional characterization of the genes and proteins was conducted using BlastKOALA.^52^ Gene Ontology (GO) information was obtained using egg-NOG mapper v2.

#### Pre-processing of illumina RNA-Seq data

Raw sequence reads obtained from Illumina RNA-Seq were trimmed with fastp^43^ (ver. 0.21.1) to remove reads with quality control below 25 or the number of unknown bases (N) per one read above 10. Trimmed reads were mapped to the HRV44^T^ genome with Bowtie2^44^ (ver. 2.3.4.3). The following process was the same as for Nanopore PCR-based RNA-Seq.

#### Cells for proteomics

HRV44^T^ was cultivated in the same manner as above. Cells grown under N_2_O-free (*t* = 0 h, n = 3) and N_2_O-added (*t* = 3, 6, and 24 h, n = 1) treatments were harvested by centrifugation at 16,000×*g* for 15 min at 4°C. Six cell pellets (approx. 1.0 x 10^9^ cells for each sample) were collected in 1.5 mL tubes and stored at -80°C.

#### Proteomics: sample prep and mass spectrometry

Bacterial cell pellets were resuspended in 200 μL of 10 mM ammonium bicarbonate with protease inhibitors (100X HALT, 1 μL). Mechanical lysis was performed (Branson 250 Sonifier; 20 kHz, 10 X 10s on ice). Samples were dried down using a speedvac to 10 μL and resuspended in 40 μL SDS lysis buffer (5% 1M TEAB, 5% SDS, 0.2% 1M MgCl_2_). The supernatant was transferred to a clean tube and proteins were quantified using the Pierce BCA Protein Assay (ThermoFisher Scientific) according to the manufacturer's instructions. Samples were reduced, alkylated, and digested directly on spin columns (PROTIFI, S-Trap micro) with the following modifications to the manufacturer's instructions. Briefly, reduction was done using dithiothreitol (DTT) to a final concentration of 20 mM, followed by incubation (10 min, 60°C). Alkylation was done with iodoacetamide (IAA) at a final concentration of 40 mM, followed by incubation (30 min, room temperature, dark). Samples were then acidified with 12% phosphoric acid to a final concentration of 1.2%, and the pH was verified to be ∼1. Binding/wash buffer (1:10, 1M TEAB: Methanol) was added to each sample, which was then processed using PROTIFI S-trap micro columns for binding and washing proteins with three rounds of the binding/wash buffer. Centrifugation (3000 x *g* for 3 min) was used for buffer passage. Additional washes were performed with methanol/chloroform (1:1 ratio) and binding/wash buffer. Proteins were digested using 1 μg Trypsin (Promega) per sample in a total volume of 20 μl and incubated for 1 hour at 47°C. Elution of the resulting peptides was performed with 50 mM TEAB followed by 50% acetonitrile with 0.2% formic acid. Peptide samples were evaporated to dryness in a speedvac (Thermo Scientific Savant) and reconstituted in 20 μL 2% acetonitrile and 0.1% formic acid.

Protein lysates were analyzed using data-dependent acquisition (DDA) proteomics (see Mudge et al.^61^). An external standard (PRTC-Peptide Retention Time Calibration Mixture; ThermoFisher, San Jose, CA) was added to monitor chromatography and MS quality, and sample run order was randomized. For each MS experiment, 1 μg of total protein and 50 fmol PRTC standard were injected and analyzed using a ThermoFisher QExactive (QE) with an EASY-nLC 1200 system. Reverse-phase chromatography was performed using an in-house packed PicoTip fused silica capillary column (40 cm x 75 μm i.d., C18 particles Dr. Maisch Reprosil) with a precolumn (3 cm x 100 μm i.d., C18 particles; Dr. Maisch Reprosil). Peptides were eluted with an acidified water-acetonitrile gradient (2% to 35% acetonitrile over 90 minutes). MS2 acquisition involved the top 20 most intense ions selected from precursor ion scans (*m*/*z* range 400-1200), with full MS data collected in centroid mode (resolution 70,000, AGC target 1 × 10^6^) and MS2 data collected at a resolution of 35,000 (centroid mode, AGC target 5 × 10^4^). MS2 ions were selected with +2, +3, and +4 charge states, and a dynamic exclusion time of 30 seconds. QC peptide mixtures, including PRTC and BSA, were injected every fifth MS experiment to monitor chromatography and MS sensitivity, with QC peptides visualized in Skyline^54^ to ensure standard peptides did not deviate more than 10%. The mass spectrometry data can be found in the ProteomeXchange Consortium via PRIDE with the data set identifier/accession PXD053214.

#### Homology search of Crp/Fnr superfamily

*Wolinella succinogenes*, the model organisms of the clade Ⅱ type *nosZ* microorganism, possess NssA, NssB, and NssC as Crp/Fnr superfamily proteins that are homologues of *Campylobacter jejuni* nitrosative stress sensing regulator NssR^27^ and regulate the expression of the several denitrification genes.^18^ Homology search and amino acid identities (AAIs) calculation of these three proteins were performed on HRV44^T^ using in silico MolecularCloning (https://www.insilico-biology.com/index.php) and BLAST (https://blast.ncbi.nlm.nih.gov/Blast.cgi), respectively. The binding site of Crp/Fnr superfamily protein (consensus sequence: TTGA-N_6_-TCAA) was searched upstream of predicted transcriptional units with up to two base mismatches permitted using in silico MolecularCloning.

### Quantification and statistical analysis

#### Transcriptome analyses

Since this is the first study to apply Nanopore PCR-based RNA-Seq to time-series transcriptome analysis of deep-sea vent chemolithoautotrophs, Illumina and Nanopore PCR-based RNA-Seq datasets were compared. In the comparison of 24 h samples, total raw counts averaged 162,061 (103,793 ∼ 269,795) and 8,852,252 (7,759,593 ∼ 10,293,742) for Nanopore PCR-based and Illumina RNA-Seq datasets, respectively. In order to evaluate the correlation of data obtained from both Nanopore PCR-based and Illumina RNA-Seq, each raw count data was normalized by counts per million (CPM) and transcripts per million (TPM). TPM value was calculated by following Equations 1 and 2.

(Equation 1) $$T_{t}=\frac{Y_{t}}{L_{t}}\times 10^{3}\;\left(Y_{t}=\text{raw}\;\text{read}\;\text{count},L_{t}=\text{gene}\;\text{length}\right)$$

(Equation 2) $${TPM}_{t}=\frac{T_{t}}{{\sum_{t}T}_{t}}\times 10^{6}$$

Pearson correlation coefficient (PCC, R) showed a positive correlation at Log_2_(CPM+4) (R = 0.57) and Log_2_(TPM+4) (R = 0.48) normalized data, respectively (Table S6). Regularized Log (rlog) transformed data showed a high correlation (R ≥ 0.78) across all gene length ranges. Positive correlations were confirmed in their Log_2_(CPM+4) and Log_2_(TPM+4) normalized data. We, therefore, decided to conduct downstream analysis using the Nanopore PCR-based RNA-Seq dataset.

For further analyses, raw count data were uploaded into iDEP.951 or .96^45^ (http://bioinformatics.sdstate.edu/idep/). Principal component analysis (PCA), differential gene expression analysis, and weighted gene co-expression network analysis (WGCNA) were performed. Count data results were transformed to Log_2_(CPM+4) with edgeR for hierarchical clustering.

Differential expression genes (DEG) (|Log_2_(fold change)| ≥ 1 and FDR < 0.05) were extracted using DESeq2.^46^ A hierarchical heatmap of DEGs was generated by the Morpheus web tool (https://software.broadinstitute.org/morpheus/) using relative expression data obtained from iDEP. Functions or families of DEGs encoding hypothetical proteins were predicted with Pfam (https://pfam.xfam.org/search). WGCNA was conducted on all CDSs with a soft threshold of 6 and minimum module size of 20 using time-course data, and the first principal component (PC1) was extracted from each color module, and a hierarchical clustering heatmap was constructed. GO enrichment analysis was performed using the R package, goseq^48^ (ver. 1.50.0), and GO terms were considered significantly enriched with an over-represented *p*-value < 0.05. REVIGO^49^ (http://revigo.irb.hr) was used to summarize enriched GO terms. *P*-value was sorted in ascending order, and top enriched GO terms were visualized using ggplot2 R package (ver. 3.4.4). For prediction of the transcriptional units and transcription start site, bam files were uploaded into Interactive Genomic Viewer (IGV) software^50^ (https://igv.org/app/), and coverage and alignment data were visualized. Furthermore, the Pearson correlation coefficient (PCC) of expression level between predicted transcriptional regulators and denitrification genes in HRV44^T^ was calculated by CPM normalized data.

#### Data analysis of proteomics

Raw mass spectrometry files were searched against a strain-specific database using Comet^55^^,^^56^ v. 2022.01 rev.0. The database included quality control sequences (enolase and PRTC) and common lab contaminants from the CRAPome.^57^ Comet search parameters included a concatenated decoy search, peptide mass tolerance of 20 ppm, 2 allowed missed cleavages, fragment bin tolerance of 0.02, and a fragment bin offset of 0. Search results were processed with PeptideProphet and ProteinProphet^58^ with a probability cut-off of 0 and then with Abacus^59^ where the false discovery rate cut-off was set at 0.01 (corresponding to a combined ProteinProphet probability of 0.64). Adjusted normalized spectral abundance factor values (ADJNSAF) were calculated in Abacus.

Proteins with ADJNSAF values of zero across all six samples were removed, and differential abundance proteins (DAPs) (|Log_2_(fold change)| ≥ 0.5 and |z-statistic| ≥ 2) were determined using QPROT^23^ and the qspec-paired command (burn-in = 2,000, iterations = 10,000, normalized = 1). Venn diagram of the DEGs and DAPs was visualized by Venny (ver. 2.1.0) (https://bioinfogp.cnb.csic.es/tools/venny/). For GO enrichment analysis of the DAPs, goseq was used with a correction of protein length. Abundance of the protein was evaluated based on Log_2_(ADJNSAF+1) values.

#### Path analysis of time-course transcriptomic data

To statistically test the effects of the N_2_O exposure and the Crp/Fnr superfamily proteins (NIL_RS03760 and NIL_RS03815) on the expression of the denitrification genes, the path analysis was performed using the lavaan^53^ package in R (ver. 0.6-13). The presence or absence of N_2_O (a categorical variable) or the duration of N_2_O exposure (a numerical variable) were used as exogenous variables. We built two models assuming each exogenous variable affects expressions of NIL_RS03760 and NIL_RS03815 and regulates expressions of each denitrification gene. Model fit was evaluated using the result of the chi-squared test, root mean squared error of approximation (RMSEA), standardized root mean square residual (SRMR), goodness of fit index (GFI), and comparative fit index (CFI) calculated by the lavaan. Standardized path coefficients were used to visualize the model structure, and the Wald *t*-test was performed to confirm the significance of each path.

## Supplemental information

Document S1. Figures S1–S10 and Tables S1–S7

Data S1. Abacus output from HRV44T cells cultured under N_2_O-added (3, 6, 24 h, n = 1) and N_2_O-free (0 h, n = 3) treatments

## References

[1] Pörtner H.-O., Roberts D.C., Tignor M., Poloczanska E.S., Mintenbeck K., Alegría A., Craig M., Langsdorf S., Löschke S., Möller V., IPCC (2022). Climate change 2022: impacts, adaptation and vulnerability. contribution of working group II to the sixth assessment report of the intergovernmental panel on climate change.

[2] Ravishankara A.R., Daniel J.S., Portmann R.W. (2009). Nitrous oxide (N_2_O): the dominant ozone-depleting substance emitted in the 21st century. Science.

[3] Tian H., Xu R., Canadell J.G., Thompson R.L., Winiwarter W., Suntharalingam P., Davidson E.A., Ciais P., Jackson R.B., Janssens-Maenhout G. (2020). A comprehensive quantification of global nitrous oxide sources and sinks. Nature.

[4] Hallin S., Philippot L., Löffler F.E., Sanford R.A., Jones C.M. (2018). Genomics and ecology of novel N_2_O-reducing microorganisms. Trends Microbiol..

[5] Jones C.M., Graf D.R.H., Bru D., Philippot L., Hallin S. (2013). The unaccounted yet abundant nitrous oxide-reducing microbial community: a potential nitrous oxide sink. ISME J..

[6] Yoon S., Nissen S., Park D., Sanford R.A., Löffler F.E. (2016). Nitrous oxide reduction kinetics distinguish bacteria harboring clade I NosZ from those harboring clade II NosZ. Appl. Environ. Microbiol..

[7] Sanford R.A., Wagner D.D., Wu Q., Chee-Sanford J.C., Thomas S.H., Cruz-García C., Rodríguez G., Massol-Deyá A., Krishnani K.K., Ritalahti K.M. (2012). Unexpected nondenitrifier nitrous oxide reductase gene diversity and abundance in soils. Proc. Natl. Acad. Sci. USA.

[8] Shan J., Sanford R.A., Chee-Sanford J., Ooi S.K., Löffler F.E., Konstantinidis K.T., Yang W.H. (2021). Beyond denitrification: the role of microbial diversity in controlling nitrous oxide reduction and soil nitrous oxide emissions. Glob. Chang. Biol..

[9] Jørgensen B.B., Boetius A. (2007). Feast and famine - microbial life in the deep-sea bed. Nat. Rev. Microbiol..

[10] Nakagawa S., Takai K., Inagaki F., Hirayama H., Nunoura T., Horikoshi K., Sako Y. (2005). Distribution, phylogenetic diversity and physiological characteristics of epsilon-*Proteobacteria* in a deep-sea hydrothermal field. Environ. Microbiol..

[11] Sievert S., Vetriani C. (2012). Chemoautotrophy at deep-sea vents, past, present, and future. Oceanography.

[12] Muto H., Takaki Y., Hirai M., Mino S., Sawayama S., Takai K., Nakagawa S. (2017). A simple and efficient RNA extraction method from deep-sea hydrothermal vent chimney structures. Microbes Environ..

[13] Mino S., Yoneyama N., Nakagawa S., Takai K., Sawabe T. (2018). Enrichment and genomic characterization of a N_2_O-reducing chemolithoautotroph from a deep-sea hydrothermal vent. Front. Bioeng. Biotechnol..

[14] Fukushi M., Mino S., Tanaka H., Nakagawa S., Takai K., Sawabe T. (2020). Biogeochemical implications of N_2_O-reducing thermophilic *Campylobacteria* in deep-sea vent fields, and the description of *Nitratiruptor labii* sp. nov. iScience.

[15] Fukazawa S., Mino S., Tsuchiya J., Nakagawa S., Takai K., Sawabe T. (2022). *Nitrosophilus kaiyonis* sp. nov., a hydrogen-sulfur- and thiosulfate-oxidizing chemolithoautotroph within “*Campylobacteria*” isolated from a deep-sea hydrothermal vent in the Mid-Okinawa Trough. Arch. Microbiol..

[16] Vollack K.U., Zumft W.G. (2001). Nitric oxide signaling and transcriptional control of denitrification genes in *Pseudomonas stutzeri*. J. Bacteriol..

[17] Bergaust L., van Spanning R.J.M., Frostegård Å., Bakken L.R. (2012). Expression of nitrous oxide reductase in *Paracoccus denitrificans* is regulated by oxygen and nitric oxide through FnrP and NNR. Microbiology.

[18] Kern M., Simon J. (2016). Three transcription regulators of the Nss family mediate the adaptive response induced by nitrate, nitric oxide or nitrous oxide in *Wolinella succinogenes*. Environ. Microbiol..

[19] Wang F., Chen Z., Pei H., Guo Z., Wen D., Liu R., Song B. (2021). Transcriptome profiling analysis of tea plant (*Camellia sinensis*) using Oxford Nanopore long-read RNA-Seq technology. Gene.

[20] Bayega A., Oikonomopoulos S., Gregoriou M.E., Tsoumani K.T., Giakountis A., Wang Y.C., Mathiopoulos K.D., Ragoussis J. (2021). Nanopore long-read RNA-seq and absolute quantification delineate transcription dynamics in early embryo development of an insect pest. Sci. Rep..

[21] Wang D., Liu X., Zhang J., Gao B., Liu P., Li J., Meng X. (2022). Identification of neuropeptides using long-read RNA-Seq in the swimming crab *Portunus trituberculatus*, and their expression profile under acute ammonia stress. Front. Physiol..

[22] Takai K., Inagaki F., Nakagawa S., Hirayama H., Nunoura T., Sako Y., Nealson K.H., Horikoshi K. (2003). Isolation and phylogenetic diversity of members of previously uncultivated ε-Proteobacteria in deep-sea hydrothermal fields. FEMS Microbiol. Lett..

[23] Choi H., Kim S., Fermin D., Tsou C.-C., Nesvizhskii A.I. (2015). QPROT: Statistical method for testing differential expression using protein-level intensity data in label-free quantitative proteomics. J. Proteomics.

[24] Adam N., Perner M. (2018). Microbially mediated hydrogen cycling in deep-sea hydrothermal vents. Front. Microbiol..

[25] Gruber T.M., Gross C.A. (2003). Multiple sigma subunits and the partitioning of bacterial transcription space. Annu. Rev. Microbiol..

[26] Körner H., Sofia H.J., Zumft W.G. (2003). Phylogeny of the bacterial superfamily of Crp-Fnr transcription regulators: exploiting the metabolic spectrum by controlling alternative gene programs. FEMS Microbiol. Rev..

[27] Elvers K.T., Turner S.M., Wainwright L.M., Marsden G., Hinds J., Cole J.A., Poole R.K., Penn C.W., Park S.F. (2005). NssR, a member of the Crp-Fnr superfamily from *Campylobacter jejuni*, regulates a nitrosative stress-responsive regulon that includes both a single-domain and a truncated haemoglobin. Mol. Microbiol..

[28] Messenger S.L., Green J. (2003). FNR-mediated regulation of *hyp* expression in *Escherichia coli*. FEMS Microbiol. Lett..

[29] Hein S., Witt S., Simon J. (2017). Clade II nitrous oxide respiration of *Wolinella succinogenes* depends on the NosG, -C1, -C2, -H electron transport module, NosB and a Rieske/cytochrome *bc* complex. Environ. Microbiol..

[30] Hein S., Simon J. (2019). Bacterial nitrous oxide respiration: electron transport chains and copper transfer reactions. Adv. Microb. Physiol..

[31] Matsubara T. (1971). Studies on denitrification: XIII. some properties of the N_2_O-anaerobically grown cell. J. Biochem..

[32] Weidenhaupt M., Rossi P., Beck C., Fischer H.-M., Hennecke H. (1996). *Bradyrhizobium japonicum* possesses two discrete sets of electron transfer flavoprotein genes: *fixA, fixB* and *etfS, etfL*. Arch. Microbiol..

[33] Jiménez-Leiva A., Cabrera J.J., Bueno E., Torres M.J., Salazar S., Bedmar E.J., Delgado M.J., Mesa S. (2019). Expanding the regulon of the *Bradyrhizobium diazoefficiens* NnrR transcription factor: new insights into the denitrification pathway. Front. Microbiol..

[34] Guttenplan S.B., Kearns D.B. (2013). Regulation of flagellar motility during biofilm formation. FEMS Microbiol. Rev..

[35] McBride M.J. (2001). Bacterial gliding motility: multiple mechanisms for cell movement over surfaces. Annu. Rev. Microbiol..

[36] Lertsethtakarn P., Ottemann K.M., Hendrixson D.R. (2011). Motility and chemotaxis in *Campylobacter* and *Helicobacter*. Annu. Rev. Microbiol..

[37] Hiis E.G., Vick S.H.W., Molstad L., Røsdal K., Jonassen K.R., Winiwarter W., Bakken L.R. (2024). Unlocking bacterial potential to reduce farmland N_2_O emissions. Nature.

[38] Perez-Riverol Y., Bai J., Bandla C., García-Seisdedos D., Hewapathirana S., Kamatchinathan S., Kundu D.J., Prakash A., Frericks-Zipper A., Eisenacher M. (2022). The PRIDE database resources in 2022: a hub for mass spectrometry-based proteomics evidences. Nucleic Acids Res..

[39] De Coster W., D’Hert S., Schultz D.T., Cruts M., Van Broeckhoven C. (2018). NanoPack: visualizing and processing long-read sequencing data. Bioinformatics.

[40] Li H. (2018). Minimap2: pairwise alignment for nucleotide sequences. Bioinformatics.

[41] Li H., Handsaker B., Wysoker A., Fennell T., Ruan J., Homer N., Marth G., Abecasis G., Durbin R., 1000 Genome Project Data Processing Subgroup (2009). The sequence alignment/map format and SAMtools. Bioinformatics.

[42] Liao Y., Smyth G.K., Shi W. (2014). featureCounts: an efficient general purpose program for assigning sequence reads to genomic features. Bioinformatics.

[43] Chen S., Zhou Y., Chen Y., Gu J. (2018). Fastp: An ultra-fast all-in-one FASTQ preprocessor. Bioinformatics.

[44] Langmead B., Salzberg S.L. (2012). Fast gapped-read alignment with Bowtie 2. Nat. Methods.

[45] Ge S.X., Son E.W., Yao R. (2018). iDEP: an integrated web application for differential expression and pathway analysis of RNA-Seq data. BMC Bioinf..

[46] Love M.I., Huber W., Anders S. (2014). Moderated estimation of fold change and dispersion for RNA-seq data with DESeq2. Genome Biol..

[47] Cantalapiedra C.P., Hernández-Plaza A., Letunic I., Bork P., Huerta-Cepas J. (2021). eggNOG-mapper v2: Functional annotation, orthology assignments, and domain prediction at the metagenomic scale. Mol. Biol. Evol..

[48] Young M.D., Wakefield M.J., Smyth G.K., Oshlack A. (2010). Gene ontology analysis for RNA-seq: accounting for selection bias. Genome Biol..

[49] Supek F., Bošnjak M., Škunca N., Šmuc T. (2011). REVIGO summarizes and visualizes long lists of gene ontology terms. PLoS One.

[50] Thorvaldsdóttir H., Robinson J.T., Mesirov J.P. (2013). Integrative Genomics Viewer (IGV): high-performance genomics data visualization and exploration. Brief. Bioinform..

[51] Aziz R.K., Bartels D., Best A.A., DeJongh M., Disz T., Edwards R.A., Formsma K., Gerdes S., Glass E.M., Kubal M. (2008). The RAST Server: Rapid annotations using subsystems technology. BMC Genom..

[52] Kanehisa M., Sato Y., Morishima K. (2016). BlastKOALA and GhostKOALA: KEGG tools for functional characterization of genome and metagenome sequences. J. Mol. Biol..

[53] Rosseel Y. (2012). Lavaan: an R package for structural equation modeling. J. Stat. Softw..

[54] MacLean B., Tomazela D.M., Shulman N., Chambers M., Finney G.L., Frewen B., Kern R., Tabb D.L., Liebler D.C., MacCoss M.J. (2010). Skyline: an open source document editor for creating and analyzing targeted proteomics experiments. Bioinformatics.

[55] Eng J.K., Jahan T.A., Hoopmann M.R. (2013). Comet: An open-source MS/MS sequence database search tool. Proteomics.

[56] Eng J.K., Hoopmann M.R., Jahan T.A., Egertson J.D., Noble W.S., MacCoss M.J. (2015). A deeper look into comet—implementation and features. J. Am. Soc. Mass Spectrom..

[57] Mellacheruvu D., Wright Z., Couzens A.L., Lambert J.P., St-Denis N.A., Li T., Miteva Y.V., Hauri S., Sardiu M.E., Low T.Y. (2013). The CRAPome: a contaminant repository for affinity purification–mass spectrometry data. Nat. Methods.

[58] Nesvizhskii A.I., Keller A., Kolker E., Aebersold R. (2003). A statistical model for identifying proteins by tandem mass spectrometry. Anal. Chem..

[59] Fermin D., Basrur V., Yocum A.K., Nesvizhskii A.I. (2011). Abacus: a computational tool for extracting and pre-processing spectral count data for label-free quantitative proteomic analysis. Proteomics.

[60] Sako Y., Takai K., Ishida Y., Uchida A., Katayama Y. (1996). *Rhodothermus obamensis* sp. nov., a modern lineage of extremely thermophilic marine bacteria. Int. J. Syst. Bacteriol..

[61] Mudge M.C., Nunn B.L., Firth E., Ewert M., Hales K., Fondrie W.E., Noble W.S., Toner J., Light B., Junge K.A. (2021). Subzero, saline incubations of *Colwellia psychrerythraea* reveal strategies and biomarkers for sustained life in extreme icy environments. Environ. Microbiol..

