# Supplementary material for: Time course transcriptomic profiling suggests Crp/Fnr transcriptional regulation of nosZ gene in a N2O-reducing thermophile
Source: iScience. 2024 Sep 30;27(11):111074. doi: 10.1016/j.isci.2024.111074 (PMC11539149; doi:10.1016/j.isci.2024.111074)
Supplement: Document S1. Figures S1–S10 and Tables S1–S7 [file mmc1.pdf]

iScience, Volume *27*

Supplemental information

Time course transcriptomic profiling suggests Crp/Fnr transcriptional regulation of *nosZ* gene in a N_2O-reducing thermophile

Jiro Tsuchiya, Sayaka Mino, Fuki Fujiwara, Nao Okuma, Yasunori Ichihashi, Robert M. Morris, Brook L. Nunn, Emma Timmins-Schiffman, and Tomoo Sawabe

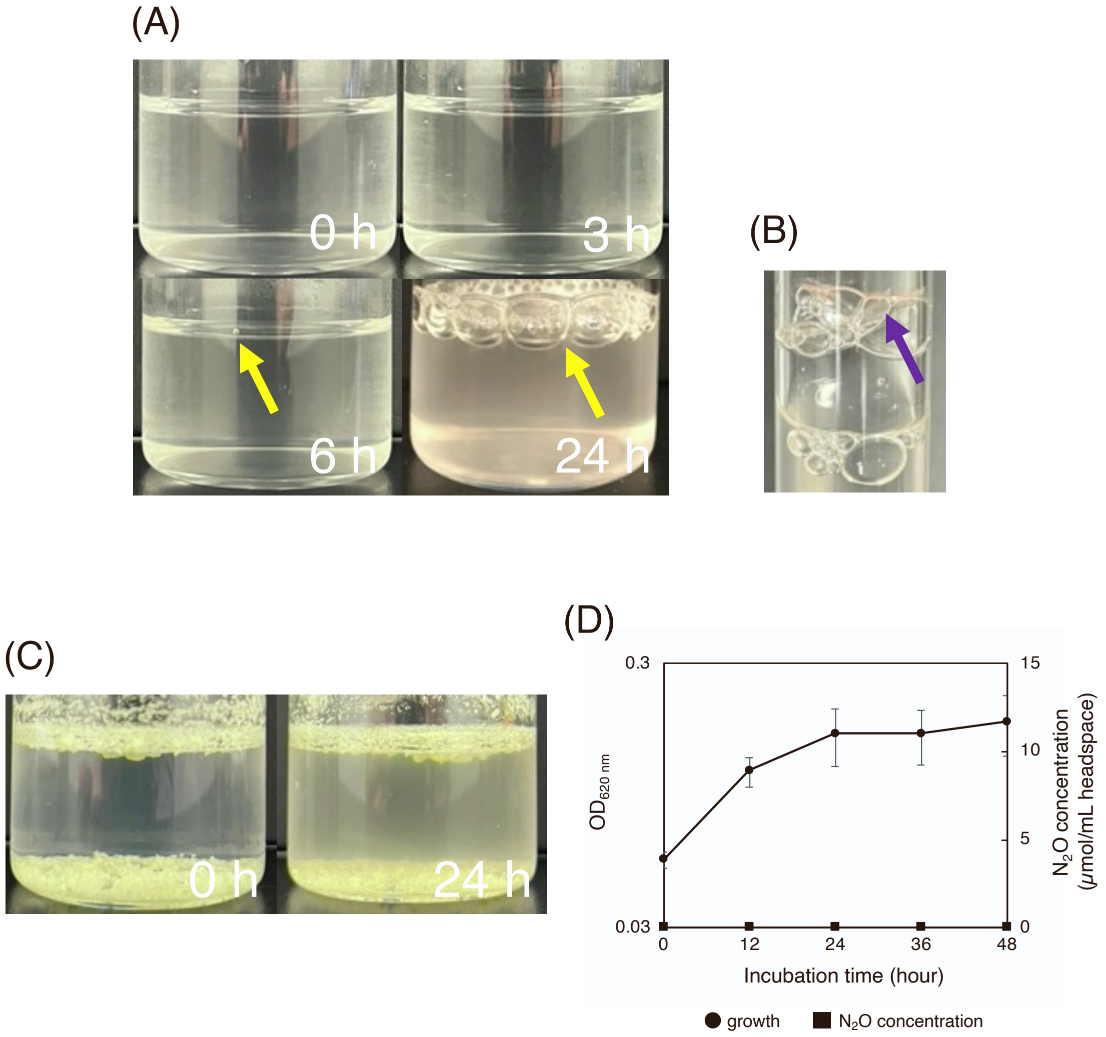

Figure S1. Images of cultures of HRV44^T under N_2O-added and MMJHS treatments. (A) Cultivation under the N_2O-added treatment in a 50 mL vial. 0 h (before the addition of N_2O) and 3, 6, and 24 h after the addition of N_2O. Yellow arrows represent visible bubbles. (B) 24 h-cultivation under the N_2O-added treatment in 15 mL test tube. Purple arrow points to the pellicle that HRV44^T produces in cultivation with N_2O. (C) Cultivation in a 50 mL vial containing MMJHS medium ($t = 0$ and 24 h). (D) Growth and headspace N_2O concentration (mean ± SD) during cultivation in MMJHS medium.

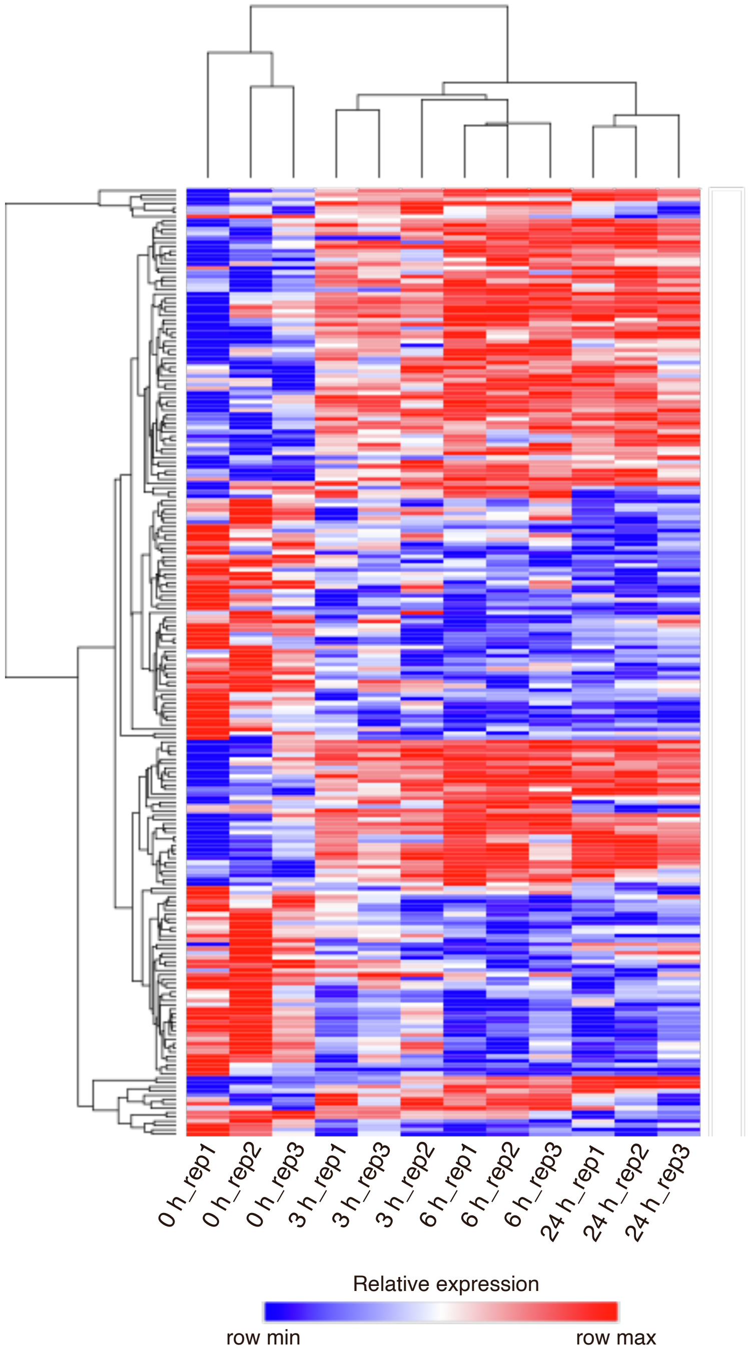

Figure S2. Hierarchical clustering heatmap of all DEGs (220 genes) in a chromosome. Clustering was conducted by Euclidean distance and average linkage. The color key represents the relative expression level: blue, lowest expression; white, intermediate expression; red, highest expression. A hierarchical heatmap was generated by the Morpheus web tool (https://software.broadinstitute.org/morpheus/).

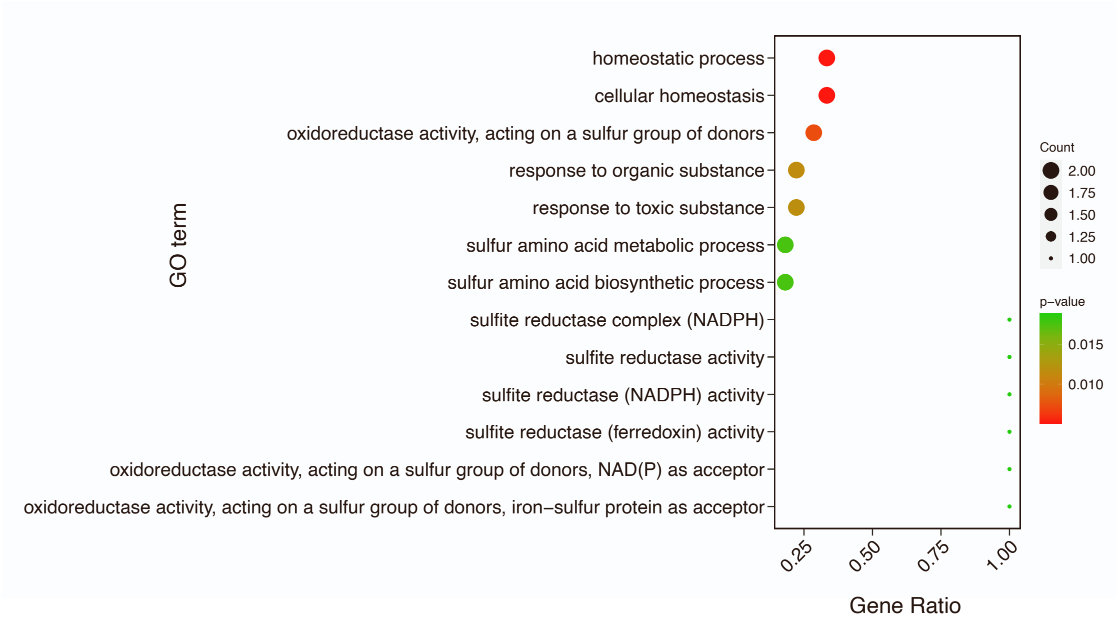

Figure S3. Integrated GO enrichment analysis of the DEGs between N_2O-added (24 h) and MMJHS (24 h) treatments. Top 10 enriched GO terms were represented.

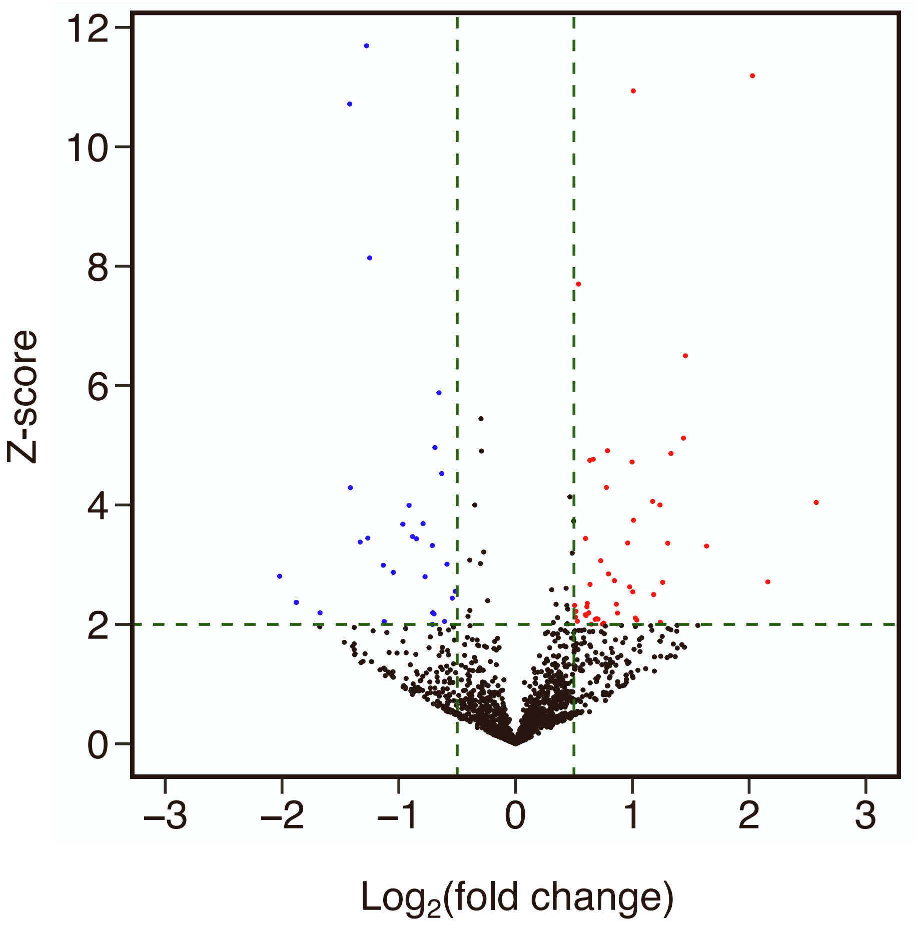

Figure S4. Proteome analyses of HRV44^{T} chromosome. Volcano plot of the relative abundance of proteins. Red and blue points represent significantly increased and decreased proteins (|Log_2FC| ≥ 0.5 and |z-statistic| ≥ 2).

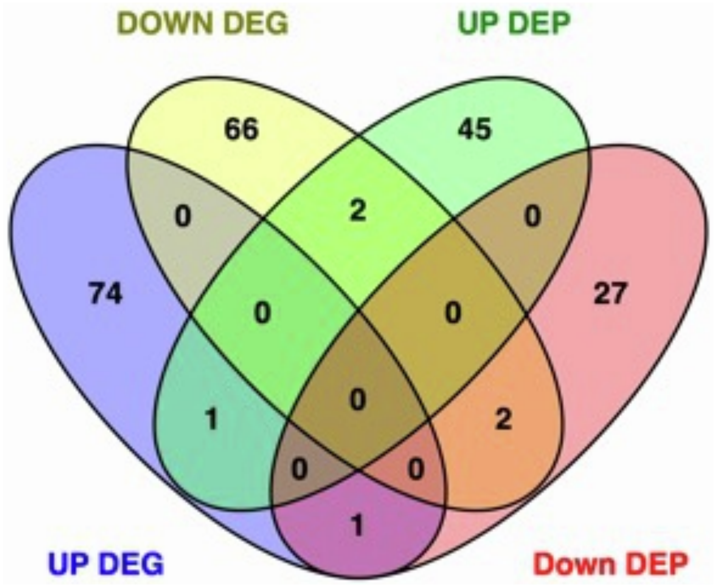

Figure S5. Venn diagram of the DEGs and DAPs in the N_2O-added treatment compared to the N_2O-free treatment. The Venn diagram was depicted by Venny 2.1 (https://bioinfogp.cnb.csic.es/tools/venny/).

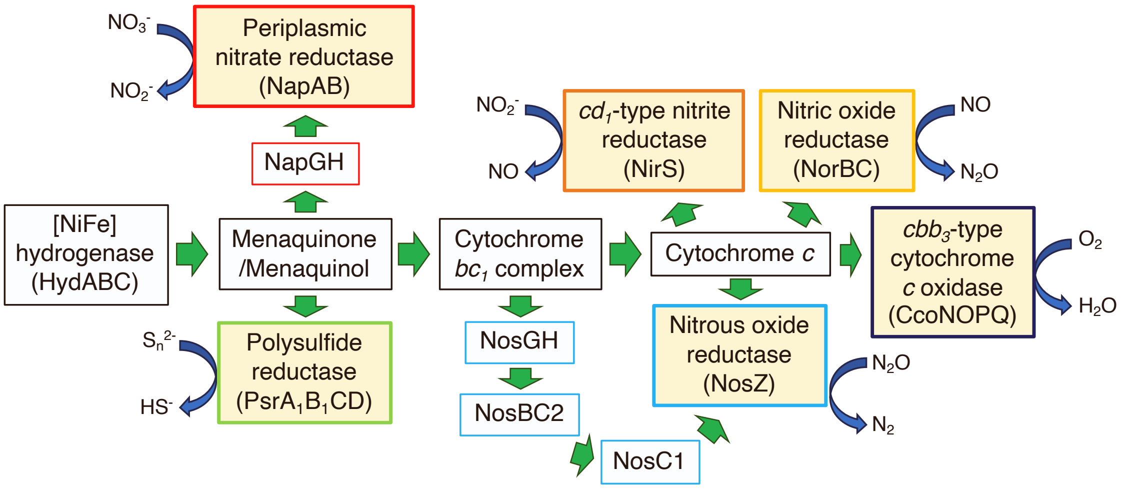

Figure S6. Electron-transport pathways to the respiration system of HRV44[T] inferred from the genome analysis. Pathways of NO_3^-, NO_2^-, NO, and N_2O reduction, sulfur reduction, and microaerobic respiration were included in this figure. Green arrows indicate electron transport pathways.

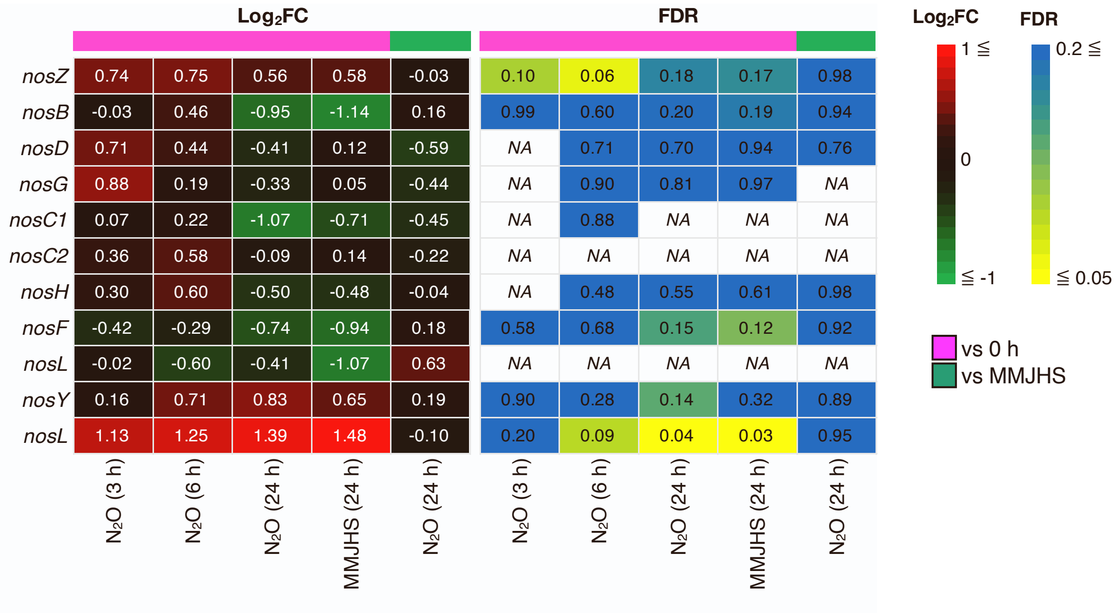

Figure S7. Log$_2$(fold change) and FDR of *nos* genes. Color panel at upside of heatmap represents a reference data in calculation of Log$_2$FC and FDR (pink, 0 h; green, MMJHS treatment). *NA*, not available in calculation of FDR by DESeq2.

(A)

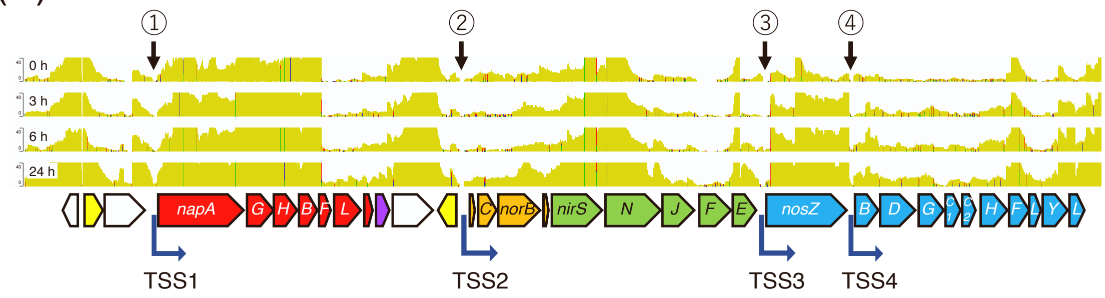

(B)

① upstream of *nap* gene cluster (396 bp)

TATTATTAAAACTGCTT**TGAATAATTATCAA**AATGTTTAGTTTAAAAAAAATCTTAGTGTTATGTTAT**ATTAATTGTATCAA**AACTAAAGCTAATACTATT
ATATAGTAGTATAAAATTTTGC**TCGATCTATTTCAA**GACTACAAATGTAATGTTAAATTGTTTTCCCATTTATCTTTCAATTTATATTTTCTTCTATTTTG
TTCTTTAAAATATATATTACTTTTAAAAATGACACAGCAATGTCACTAATAAAGACATAAATAATCTTAATTATTTTAATAACTATATTTTA**TTATTTTGACT**
CAAGTCAAGGCGAGGAGTTTAGAAAATCTATAAAATTTTTTC**G**TTAATTCACAATTGCCGAGTTACGGCAAAATCTTATG**AGGAGG**CATAT-***napA***

② upstream of *nor* gene cluster (287 bp)

CTTATCCTCTATTTTTTTAATAAATTATATAACTATTTACTTAAAAAAAAACTATCATTTTAAAATTCCAATAAATTTTGCAAAGAAAT**ATTAAAAATATCA**
ATAAATTTTACCTATTGTGTGACTTAATTGTGACATAAAAATAAAACTAATT**TTAATTATTAACAA**AAAAGGTTATTTTTTTAAGTTTT**TTGACTAATGT**
CAAAGAAAAATGTTCTATCAAATTATAAAATTTACT**A**TAACTTTTGGTTTAAAGGTTGTATTAAACCTAAATTA**AGGAGG**AATAG-***norE***

③ upstream of *nosZ* (309 bp)

ACTAGATTTAAAATTTAAGCATAATATAGTCAAAAAAGGTTTCTTCTATAAGCT**TACATAAGTCTCAA**TATTTTCTACAAATGCCATAAAATTGTTAAT**T**
TTATAAGAGTTAAAAAATAA**TTAACGAAAGTCAC**ACAAATATAATATACCGTTTTATTTTT**TTAAGAATATTCAA**TTTTTTTTAAATAAAAATTATAATA
CATATAATAATAACCAAAAAAAATACTTTCAAAACTTAATAAGTTAATATATATTTAAGTGTTTAGGAATTATAA**TTGAATTAACACTA**ATTTAACAA**AGGA**
GGCAAAAT-***nosZ***

④ upstream of *nosB* (206 bp)

TCATAAGAGGGGGACTCCCCCTCATTTTCCTCATATGATTTACGTTTGTCTTTAGTAATATTTTAATAACAAAATATAACACTCTCTTATAAATATTAGC
TGAAGTTATTATAAAGATAAATGTAAATTATAT**TTGATTATAATCAA**TATATAAAACAAGAAAAGAGGGTATAA**TTGATAATTATGAAAAACATAAAGG**
AAGCCTT-***nosB***

Figure S8. Prediction of transcriptional regulation of denitrification genes. (A) Visualized coverage data. Black arrows represent the non-coding regions where the number of the mapped reads was remarkably dropped. TSS, predicted transcript start site. (B) Non-coding sequences upstream of the predicted transcriptional units. Red represents predicted TSS in N_2O-added samples (24 h); light green, Ribosome Binding Site (RBS); yellow, predicted Crp/Fnr transcriptional regulator binding site (underlined: mismatch bases up to 2 bases).

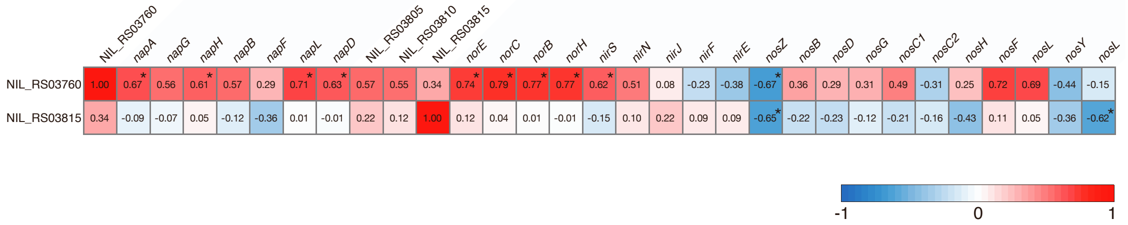

Figure S9. Co-expression relation between Crp/Fnr superfamily protein (NIL_RS03760 and NIL_RS03815) and denitrification genes. Values represent Pearson correlation coefficient (*$p < 0.05$).

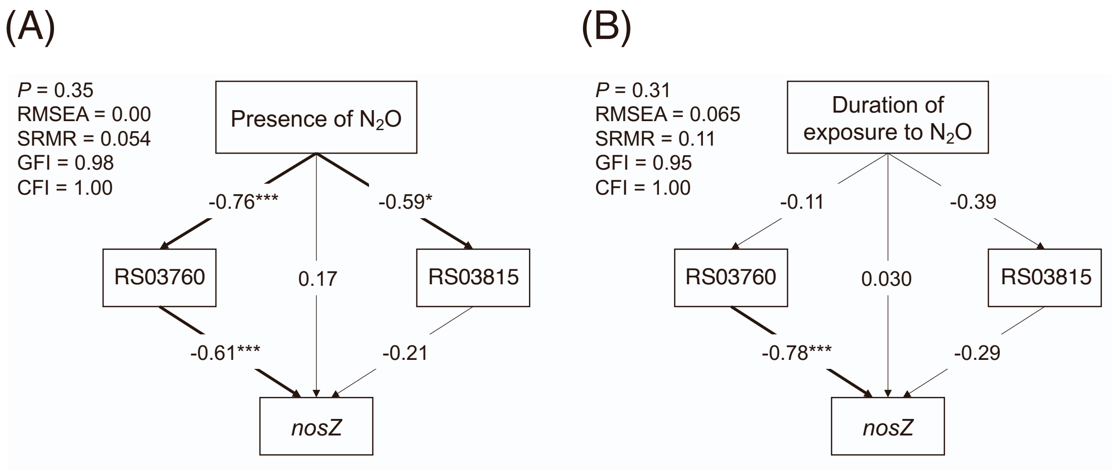

Figure S10. Path diagram showing the effects of N_2O addition and the Crp/Fnr superfamily proteins (NIL_RS03760 and RS03815) on the expression of *nosZ*. (A) The presence of N_2O or (B) the duration of the exposure to N_2O was included as exogeneous variable in the model. Bold arrows are significant paths (p < 0.05), and values at arrows are standardized path coefficients. Significance: *p<0.05, **p<0.01, ***p<0.005.

Table S1. DEGs in N_2O-added (24 h) treatment compared to MMJHS treatment

Gene ID	Log_2FC	Gene	Annotation
**Up-regulated DEGs in N_2O-added (24 h) treatment (21 genes)**			
NIL_RS00205	1.21		DUF505 domain-containing protein
NIL_RS00210	1.02	*ahpC*	Peroxiredoxin Bcp-type
NIL_RS00215	1.18		DUF308 domain-containing protein
NIL_RS00220	1.58		YfdX family protein
NIL_RS00300	1.36		Phosphoribosylaminoimidazole synthetase
NIL_RS00830	2.46	*cysI*	Nitrite/sulfite reductase
NIL_RS01075	1.01		Cytochrome c family protein
NIL_RS01080	1.40		Two-component transcriptional response regulator OmpR family
NIL_RS01085	1.24	*barA*	ATP-binding protein
NIL_RS01470	1.38		Transcriptional regulator LysR family
NIL_RS02055	1.05		Hpt domain-containing protein
NIL_RS02575	1.27	*ylxR*	DUF448 domain-containing protein
NIL_RS02855	1.23	*groES*	Heat shock protein 10 kDa family chaperone GroES
NIL_RS03340	1.11	*hrcA*	Heat-inducible transcription repressor HrcA
NIL_RS04400	1.01	*hup*	HU family DNA-binding protein
NIL_RS05095	2.68		FixH family protein
NIL_RS05100	2.97	*crdB*	TolC family protein
NIL_RS05535	1.09	*fieF*	Ferrous-iron efflux pump FieF
NIL_RS05765	1.85		BrnT family toxin
NIL_RS06505	1.67	*fliA*	RNA polymerase sigma factor for flagellar operon
NIL_RS07855	4.07		AlpA family phage regulatory protein
**Down-regulated DEGs in N_2O-added (24 h) treatment (18 genes)**			
NIL_RS00620	-1.81	*nifU*	Iron-sulfur cluster assembly scaffold protein
NIL_RS00625	-2.25	*nifS*	Cysteine desulfurase
NIL_RS01255	-2.24		Hemerythrin domain-containing protein
NIL_RS01865	-1.22	*prmA*	50S ribosomal protein L11 methyltransferase
NIL_RS02125	-1.36	*atpE*	F_0F_1 ATP synthase subunit C
NIL_RS02865	-1.54		Hypothetical protein
NIL_RS02975	-1.52		IS110 family transposase
NIL_RS03015	-1.03	*ccsA*	Cytochrome c biogenesis protein CcsA
NIL_RS03170	-4.80		Hypothetical protein
NIL_RS03175	-4.59		Hypothetical protein
NIL_RS05410	-1.35	*fapR*	Hot dog fold protein HP0420 / Methylthioribose-1-phosphate isomerase
NIL_RS05445	-1.38		EAL domain-containing protein
NIL_RS05450	-1.42	*phoB*	Phosphate regulon transcriptional regulatory protein PhoB
NIL_RS06610	-1.36	*mrp*	Mrp/NBP35 family ATP-binding protein
NIL_RS07095	-1.46		IS110 family transposase
NIL_RS09050	-1.79	*murB*	UDP-N-acetylenolpyruvoylglucosamine reductase
NIL_RS09055	-1.29	*tpx*	thiol peroxidase
NIL_RS09935	-1.36	*cbiK*	DUF4198 domain-containing protein

Table S2. DAPs list in comparison of N_2O-free and N_2O-added treatments.

(A) The increased DAPs. (B) The decreased DAPs.

(A)

Protein ID	Log_2FC	|z-statistic|	Protein	Annotation
WP_187646885.1	2.575	4.0405	NrdD	Ribonucleotide reductase of class III (anaerobic) large subunit
WP_187647101.1	2.16	2.7112	PolX	DNA polymerase X family/PHP domain protein
WP_187648295.1	2.029	11.1896	NirS	Nitrite reductase
WP_187648232.1	1.636	3.3109		Hypothetical protein
WP_187647082.1	1.455	6.4987	EtfA	Electron transfer flavoprotein alpha subunit
WP_187647083.1	1.438	5.1192	Etf	Electron transfer flavoprotein-ubiquinone oxidoreductase
WP_187646823.1	1.331	4.8619		Hypothetical protein
WP_246434421.1	1.303	3.3592	Mdh	Malate dehydrogenase
WP_187647559.1	1.26	2.7035		COG1180: Radical SAM Pyruvate-formate lyase-activating enzyme like
WP_187648304.1	1.24	2.0345	NosC2	c-type cytochrome
WP_187648292.1	1.237	4.0005	NorC	Nitric-oxide reductase subunit C
WP_187648492.1	1.183	2.4988		Metallo-beta-lactamase family protein RNA-specific
WP_187648114.1	1.174	4.0601		NosL-like protein
WP_187647217.1	1.038	2.0716		17 kDa surface antigen
WP_187647700.1	1.026	2.1056	AldB	Aldehyde dehydrogenase
WP_187646900.1	1.01	3.7446		Hypothetical protein
WP_187648282.1	1.008	10.9362	NapA	Periplasmic nitrate reductase
WP_187648039.1	1.004	2.5442	CheB	Chemotaxis response regulator protein-glutamate methylesterase CheB
WP_187647133.1	0.998	4.7192		Hypothetical protein
WP_187648440.1	0.977	2.6274		Hypothetical protein
WP_187648490.1	0.96	3.3626	PpsA	Phosphoenolpyruvate synthase
WP_187647472.1	0.873	2.1891		Hypothetical protein
WP_187648427.1	0.863	2.3378	PdxJ	Pyridoxine 5'-phosphate synthase
WP_187648284.1	0.847	2.7319	NapB	Nitrate reductase cytochrome c_{550}-type subunit
WP_187648738.1	0.796	2.8447		N-acetyltransferase
WP_187646944.1	0.787	4.9073	ThiJ	DJ-1/YajL/PfpI superfamily includes chaperone protein YajL (former ThiJ) parkinsonism-associated protein DJ-1 peptidases PfpI Hsp31
WP_187646977.1	0.778	4.2923	FlaA	Flagellin protein FlaA
WP_246434469.1	0.755	2.0203	FtsK	DNA translocase FtsK
WP_187647971.1	0.745	2.0041	NusB	Transcription termination protein NusB
WP_187648120.1	0.729	3.0662		Hypothetical protein
WP_187647471.1	0.707	2.0871		Hypothetical protein
WP_187647398.1	0.694	2.0931	PorD	Pyruvate:ferredoxin oxidoreductase delta subunit
WP_187647018.1	0.682	2.0834	CheY	Chemotaxis regulator - transmits chemoreceptor signals to flagellar motor components CheY
WP_187647733.1	0.665	4.7663	SqrB	Sulfide:quinone oxidoreductase Type II
WP_187648054.1	0.648	2.0029		Methyl-accepting chemotaxis sensor/transducer protein
WP_187647338.1	0.638	2.6694	GabD	Aldehyde dehydrogenase
WP_187648045.1	0.636	4.7468	AcsA	Acetyl-CoA synthetase
WP_187647749.1	0.627	2.1928		N-acetyltransferase
WP_187647479.1	0.614	2.3507	AlgA	Mannose-1-phosphate guanylyltransferase / Mannose-6-phosphate isomerase
WP_187648078.1	0.611	2.2947		Cytochrome c-type protein
WP_187647437.1	0.604	2.1478	AccA	Biotin carboxylase of acetyl-CoA carboxylase
WP_187647415.1	0.599	3.438	OprC	TonB-dependent receptor
WP_187646874.1	0.598	2.1591	HdrB	Heterodisulfide reductase subunit B-like protein
WP_187648289.1	0.539	7.7005		Hypothetical protein
WP_187646767.1	0.529	2.0528	TrpD	Anthranilate phosphoribosyltransferase
WP_187647328.1	0.517	2.2179	ArgS	Arginyl-tRNA synthetase
WP_187648393.1	0.512	2.1226	Hup	DNA-binding protein HU
WP_187647142.1	0.506	2.3182	HisD	Histidinol dehydrogenase

Table S2. continued

(B)

Protein ID	Log_2FC	|z-statistic|	Protein	Annotation
WP_187647514.1	-0.517	2.5541	RplD	LSU ribosomal protein L4p (L1e)
WP_187647787.1	-0.542	2.4384	RplK	LSU ribosomal protein L11p (L12e)
WP_187647543.1	-0.587	3.0084		Hypothetical protein
WP_197972071.1	-0.608	2.0487	ClpA	ATP-dependent Clp protease ATP-binding subunit ClpA
WP_187647503.1	-0.632	4.5246	RplE	LSU ribosomal protein L5p (L11e)
WP_187647203.1	-0.656	5.8782	AhpC	Alkyl hydroperoxide reductase subunit C-like protein
WP_187647788.1	-0.69	4.9619	RplA	LSU ribosomal protein L1p (L10Ae)
WP_187646908.1	-0.698	2.1749		Hypothetical protein
WP_187647811.1	-0.709	2.1935	RbpA	RNA-binding protein
WP_187648657.1	-0.712	2.0006		Type-F conjugative transfer system secretin TraK
WP_187647737.1	-0.713	3.3201	NifS	Cysteine desulfurase
WP_187647508.1	-0.775	2.7988	RplP	LSU ribosomal protein L16p (L10e)
WP_187647722.1	-0.792	3.688	CcpA	Cytochrome c_{551} peroxidase
WP_187647068.1	-0.848	3.4324	RplU	LSU ribosomal protein L21p
WP_187647060.1	-0.882	3.4707		Hypothetical protein
WP_187647498.1	-0.912	3.9949	RpsE	SSU ribosomal protein S5p (S2e)
WP_187646876.1	-0.965	3.6785	SorA	Superoxide reductase
WP_187647824.1	-1.046	2.872	FtsH	Cell division-associated ATP-dependent zinc metalloprotease FtsH
WP_187648649.1	-1.125	2.0476		Conjugal transfer protein TraH
WP_187647316.1	-1.133	2.9908	MiaB	tRNA-i(6)A37 methylthiotransferase
WP_187647411.1	-1.249	8.1387	CcoO	Cytochrome c oxidase (cbb_3-type) subunit CcoO
WP_187647793.1	-1.265	3.4444	RpsL	SSU ribosomal protein S12p (S23e)
WP_187647451.1	-1.276	11.6917	Tpx	Thiol peroxidase Tpx-type
WP_187647606.1	-1.331	3.378	HybP	Hydrogenase maturation protease
WP_187647019.1	-1.414	4.289		Methyl-accepting chemotaxis sensor/transducer protein
WP_187647321.1	-1.42	10.7163		Hypothetical protein
WP_187648017.1	-1.674	2.1942	YpdF	Aminopeptidase YpdF (MP- MA- MS- AP- NP- specific)
WP_187647755.1	-1.876	2.3691		Hypothetical protein
WP_187648013.1	-1.879	2.3676		Hypothetical protein
WP_187647465.1	-2.02	2.8065	DeaD	DEAD-box ATP-dependent RNA helicase DeaD (CshA)

Table S3. Pearson correlation coefficient (PCC) between transcriptome and proteome data normalized to Log_2(CPM+1) and Log_2(ADJNSAF+1), respectively. PCC was calculated using data sets of 1,316 genes.

		Proteomics					
	PCC	0 h-1	0 h-2	0 h-3	3 h	6 h	24 h
Transcriptomics	0 h-1	0.23	0.24	0.26	0.25	0.23	0.26
	0 h-2	0.27	0.29	0.31	0.29	0.28	0.27
	0 h-3	0.23	0.27	0.28	0.26	0.24	0.23
	3 h-1	0.24	0.26	0.27	0.25	0.25	0.24
	3 h-2	0.25	0.28	0.29	0.27	0.26	0.26
	3 h-3	0.25	0.28	0.29	0.27	0.27	0.25
	6 h-1	0.26	0.29	0.30	0.28	0.28	0.26
	6 h-2	0.25	0.28	0.29	0.27	0.27	0.24
	6 h-3	0.24	0.27	0.28	0.26	0.25	0.23
	24 h-1	0.29	0.31	0.33	0.31	0.30	0.29
	24 h-2	0.24	0.27	0.27	0.26	0.24	0.24
	24 h-3	0.21	0.24	0.25	0.23	0.22	0.21

Table S4. DEGs related to Crp/Fnr superfamily proteins and motility. Log_2(fold change) written by blue represent significant down-regulated expression (|Log_2(fold change)| ≥ 1 and FDR < 0.05). n.d. means “not detected”.

Gene ID	WGCNA module	Log_2FC (vs N_2O-free treatment, 0 h)				Gene	Annotation
		N_2O (3 h)	N_2O (6 h)	N_2O (24 h)	MMJHS		
**Crp/Fnr superfamily protein**							
NIL_RS03760	yellow	-1.14	-1.60	-0.72	-2.02		Crp/Fnr superfamily protein
**Motility**							
NIL_RS03080	n.d.	-0.76	-0.63	-1.08	-1.24	*pilT*	Twitching motility protein PilT
NIL_RS06355	black	-0.71	-0.93	-1.33	-0.80	*flgK*	Flagellar hook-associated protein FlgK
NIL_RS06455	brown	-1.25	-1.12	-1.24	-1.43	*flgE*	Flagellar hook protein FlgE
NIL_RS06460	yellow	-1.23	-1.56	-1.25	-2.36	*flgD*	Flagellar basal-body rod modification protein FlgD
NIL_RS06490	black	-1.12	-1.35	-1.21	-1.73	*flgB*	Flagellar basal-body rod protein FlgB
NIL_RS06505	purple	-0.70	-1.05	-0.09	-1.77	*fliA*	RNA polymerase sigma factor for flagellar operon
NIL_RS06565	pink	-0.71	-1.18	-0.84	-1.51	*fliG*	Flagellar motor switch protein FliG

Table S5. Amino acid identity (AAI) and query cover (QC) between Crp/Fnr superfamily proteins in strain HRV44^T and *W. succinogenes* DSM 1740

			W. succinogenes DSM 1740		
			NssA	NssB	NssC
HRV44^T	NIL_RS03760	AAI	44.8%	29.2%	26.1%
		QC	100%	91%	95%
	NIL_RS03815	AAI	33.3%	50.0%	28.2%
		QC	88%	94%	84%

Table S6. Correlation comparison by gene length in Log_2(CPM+4), Log_2(TPM+4), and rlog transformed data between Nanopore PCR-based and Illumina RNA-Seq datasets. PCC represents the Pearson correlation coefficient.

Gene length (bp)	The number of gene	PCC (R)		
		Log_2(CPM+4)	Log_2(TPM+4)	rlog
total	2,056	0.57	0.48	0.83
≤ 500	623	0.54	0.51	0.83
500 ～ 1,000	742	0.51	0.51	0.78
1,000 ～ 1,500	427	0.49	0.47	0.78
1,500 ～ 2,000	153	0.60	0.61	0.84
> 2,000	111	0.61	0.59	0.84

Table S7. Sequencing statistic of Nanopore PCR-based RNA-Seq

strain	condition	sample	N_2O	barcode	num_seqs	sum_len	min_len	avg_len	max_len	total raw count
HRV44^T	H_2-O_2 (0.1%, *v*/*v*)	0 h_rep1	free	1	820,197	366,848,612	83	447	4,315	15,065
HRV44^T	H_2-O_2 (0.1%, *v*/*v*)	0 h_rep2	free	2	725,360	356,511,026	90	492	4,463	31,444
HRV44^T	H_2-O_2 (0.1%, *v*/*v*)	0 h_rep3	free	3	722,043	353,875,991	91	490	5,974	51,678
HRV44^T	H_2-N_2O	3 h_rep1	added	4	911,514	389,424,423	86	427	6,157	51,463
HRV44^T	H_2-N_2O	3 h_rep2	added	5	820,227	397,075,857	88	484	4,187	28,955
HRV44^T	H_2-N_2O	3 h_rep3	added	6	684,379	381,499,967	94	557	5,870	47,389
HRV44^T	H_2-N_2O	6 h_rep1	added	7	959,776	427,771,906	88	446	5,026	73,138
HRV44^T	H_2-N_2O	6 h_rep2	added	8	1,004,667	442,947,460	90	441	4,824	56,270
HRV44^T	H_2-N_2O	6 h_rep3	added	9	1,018,897	454,405,712	84	446	5,332	56,016
HRV44^T	H_2-N_2O	24 h_rep1	added	10	1,102,049	501,457,418	80	455	4,550	93,121
HRV44^T	H_2-N_2O	24 h_rep2	added	11	868,672	406,949,536	84	469	4,721	84,478
HRV44^T	H_2-N_2O	24 h_rep3	added	12	1,187,904	573,772,707	84	483	6,003	214,003
HRV44^T	MMJHS	MMJHS_rep1	free	1	962,757	501,534,113	118	520.9	19,868	64,101
HRV44^T	MMJHS	MMJHS_rep2	free	2	840,880	460,574,490	98	547.7	10,275	64,476
HRV44^T	MMJHS	MMJHS_rep3	free	3	854,860	438,856,325	135	513.4	9,917	66,772
